# Combining full-length gene assay and SpliceAI to interpret the splicing impact of all possible *SPINK1* coding variants

**DOI:** 10.1186/s40246-024-00586-9

**Published:** 2024-02-27

**Authors:** Hao Wu, Jin-Huan Lin, Xin-Ying Tang, Gaëlle Marenne, Wen-Bin Zou, Sacha Schutz, Emmanuelle Masson, Emmanuelle Génin, Yann Fichou, Gerald Le Gac, Claude Férec, Zhuan Liao, Jian-Min Chen

**Affiliations:** 1 Department of Gastroenterology, Changhai Hospital, Naval Medical University, 168 Changhai Road, Shanghai, 200433 China; 2 Shanghai Institute of Pancreatic Diseases, Shanghai, China; 3 Department of Prevention and Health Care, Eastern Hepatobiliary Surgery Hospital, Naval Medical University, Shanghai, China; 4 Univ Brest, Inserm, EFS, UMR 1078, GGB, F-29200 Brest, France; 5 Service de Génétique Médicale et de Biologie de La Reproduction, CHRU Brest, Brest, France

**Keywords:** Chronic pancreatitis, Full-length gene splicing assay (FLGSA), Precision medicine in genetics, Pre-mRNA splicing, Single-nucleotide variants (SNVs), SpliceAI, Splicing prediction algorithms, Splice site, *SPINK1* gene, Variant interpretation

## Abstract

**Background:**

Single-nucleotide variants (SNVs) within gene coding sequences can significantly impact pre-mRNA splicing, bearing profound implications for pathogenic mechanisms and precision medicine. In this study, we aim to harness the well-established full-length gene splicing assay (FLGSA) in conjunction with SpliceAI to prospectively interpret the splicing effects of all potential coding SNVs within the four-exon *SPINK1* gene, a gene associated with chronic pancreatitis.

**Results:**

Our study began with a retrospective analysis of 27 *SPINK1* coding SNVs previously assessed using FLGSA, proceeded with a prospective analysis of 35 new FLGSA-tested *SPINK1* coding SNVs, followed by data extrapolation, and ended with further validation. In total, we analyzed 67 *SPINK1* coding SNVs, which account for 9.3% of the 720 possible coding SNVs. Among these 67 FLGSA-analyzed SNVs, 12 were found to impact splicing. Through detailed comparison of FLGSA results and SpliceAI predictions, we inferred that the remaining 653 untested coding SNVs in the *SPINK1* gene are unlikely to significantly affect splicing. Of the 12 splice-altering events, nine produced both normally spliced and aberrantly spliced transcripts, while the remaining three only generated aberrantly spliced transcripts. These splice-impacting SNVs were found solely in exons 1 and 2, notably at the first and/or last coding nucleotides of these exons. Among the 12 splice-altering events, 11 were missense variants (2.17% of 506 potential missense variants), and one was synonymous (0.61% of 164 potential synonymous variants). Notably, adjusting the SpliceAI cut-off to 0.30 instead of the conventional 0.20 would improve specificity without reducing sensitivity.

**Conclusions:**

By integrating FLGSA with SpliceAI, we have determined that less than 2% (1.67%) of all possible coding SNVs in *SPINK1* significantly influence splicing outcomes. Our findings emphasize the critical importance of conducting splicing analysis within the broader genomic sequence context of the study gene and highlight the inherent uncertainties associated with intermediate SpliceAI scores (0.20 to 0.80). This study contributes to the field by being the first to prospectively interpret all potential coding SNVs in a disease-associated gene with a high degree of accuracy, representing a meaningful attempt at shifting from retrospective to prospective variant analysis in the era of exome and genome sequencing.

**Supplementary Information:**

The online version contains supplementary material available at 10.1186/s40246-024-00586-9.

## Background

Single-nucleotide variants (SNVs) within the coding sequences of genes have the potential to exert a profound influence on pre-mRNA splicing. Remarkably, approximately 22% of disease-associated missense variants have been recognized as having the capacity to modulate pre-mRNA splicing [1, 2]. This influence goes beyond missense variants and includes synonymous and nonsense variants [3, 4]. These findings have far-reaching implications for our understanding of disease pathogenesis and the advancement of precision medicine. For instance, what was once considered a 'neutral' missense variant or a 'synonymous' variant may, upon closer examination, be found to be disease-causing or related due to its impact on splicing. Similarly, the effectiveness of molecular treatment strategies targeting specific 'missense' or 'nonsense' variants may be compromised if these variants unexpectedly affect splicing.

The gold standard for studying the splicing effects of clinically detected SNVs is the analysis of RNA from pathophysiologically relevant tissues. However, practical constraints often limit access to these tissue samples [5]. As an alternative, RNA analysis from patient blood cells or immortalized lymphoblastoid cells is commonly employed, under the assumption that the gene of interest exhibits normal expression in these cell types [6]. When these options prove unfeasible, the frequently employed approach is the cell culture-based minigene splicing assay [7]. It is essential to acknowledge the inherent limitation of this assay – its inability to capture the broader genomic context of the study gene. This limitation could lead to erroneous findings [8, 9] due to the intricate nature of splicing regulation [10, 11].

In recent years, significant progress has been made in predicting the splicing outcomes of SNVs. An notable development is SpliceAI [12], a 32-layer deep neural network, which, since its introduction, has become a widely utilized tool in medical genetics for predicting splicing variants (e.g., [13–19]). While these in silico prediction tools are valuable, they cannot be used in isolation to establish pathogenicity in accordance with variant classification guidelines recommended by the American College of Medical Genetics and Genomics (ACMG) [20]. Instead, they serve as first-line tools for variant classification and prioritization.

Another critical issue in medical genetics lies in the retrospective nature of functional analyses conducted on clinically identified variants [21]. With exome and genome sequencing becoming commonplace in clinical diagnostics, the urgency for prompt functional analysis is ever-increasing. Typically conducted in specialized labs, these analyses are vital for accurate variant classification [20]. However, the traditional, retrospective approach struggles to meet the rapidly evolving demands of precision medicine. Addressing this challenge requires a fundamental shift from retrospective to prospective assessment, examining the functional impact of all potential SNVs at clinically significant loci in the human genome [21, 22]. The development of multiplexed assays for variant effects (MAVE) has catalyzed this shift, enabling the collection of functional data for a vast array of variants in a single experiment [23]. A notable example is the prospective assessment of the functional impact, including splicing, of nearly 4,000 single nucleotide substitutions across 13 exons of the 23-exon *BRCA1* gene (NM_007294.3) [24]. However, MAVE is technically and resource demanding, limiting its widespread application in many laboratories.

*SPINK1* (OMIM #167790) stands out as one of the primary genes associated with chronic pancreatitis [25–28]. Located on chromosome 5q32, the pathologically relevant *SPINK1* mRNA isoform (NM_001379610.1) comprises four exons, encoding a 79-amino acid precursor protein that eventually yields the mature 56-amino-acid pancreatic secretory trypsin inhibitor [29, 30]. Loss-of-function variants in the *SPINK1* gene increase susceptibility to chronic pancreatitis through the trypsin-dependent pathway [25, 31, 32]. Previously, we successfully cloned the ~ 7-kb genomic sequence of the four-exon *SPINK1* gene into the pcDNA3.1/V5-His-TOPO vector, establishing a cell culture-based full-length gene splicing assay (FLGSA) [33]. Notably, FLGSA, unlike the frequently used minigene assay, preserves the broader natural genomic context of the gene under investigation—a crucial factor considering the intricacies of splicing regulation. Naturally, FLGSA also provides a practical advantage over the minigene assay, enabling comprehensive analysis of all coding and intronic variants within a consistent genomic framework.

In the context of the *SPINK1* gene, we have previously employed the FLGSA assay to analyze both known coding and intronic variants [8, 34–38]. The accuracy of the FLGSA assay is illuminated by the study of the *SPINK1* c.194 + 2 T > C variant, a type of variant often considered to cause a complete functional loss of the affected allele due to its occurrence within the canonical GT splice donor site [39]. Specifically, the findings from the FLGSA assay [34] were in alignment with in vivo splicing data [40] for c.194 + 2 T > C, revealing a notable presence of wild-type (WT) transcripts alongside with exon 3-skipping aberrant transcripts (N.B. the ratio of WT transcripts to aberrant transcripts was subsequently estimated to be 1:9 [41]). Remarkably, this preservation of 10% residual function correlates with the less severe phenotypes observed in *SPINK1* c.194 + 2 T > C homozygotes, who exhibit chronic pancreatitis with variable expressivity [42]. In contrast, homozygous *SPINK1* variants leading to a total loss (100%) of the gene product are associated with a more severe phenotype referred to as severe infantile isolated exocrine pancreatic insufficiency [43].

In this study, we set out to harness the combined power of the FLGSA assay and SpliceAI's predictive capabilities to prospectively interpret the splicing effects of all potential coding SNVs within the *SPINK1* gene. The preprint of this manuscript is available on medRxiv [44].

## Methods

### Research rationale and strategy

**Fig. 1:**
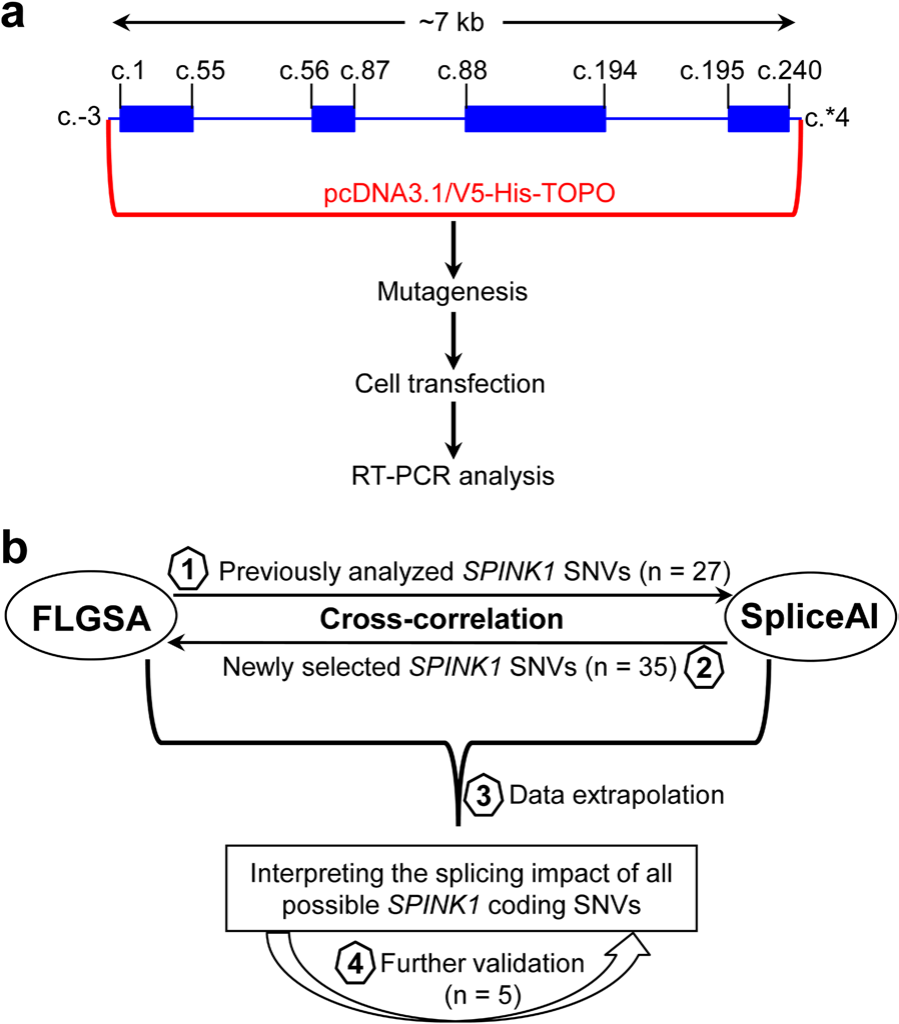
Overview of the FLGSA assay and research strategy. **a** Representation of the *SPINK1* full-length gene expression vector and the experimental steps involved in the FLGSA assay for each study variant. The coding sequences of the four-exon *SPINK1* gene are depicted to scale, while the intronic and untranslated region sequences are not. The reference *SPINK1* genomic sequence is NG_008356.2, and the reference *SPINK1* mRNA sequence is MANE (Matched Annotation from the NCBI and EMBL-EBI [45]) select ENST00000296695 or NM_001379610.1. NM_001379610.1 represents the *SPINK1* transcript isoform expressed in the exocrine pancreas [29, 30]. The starting and ending positions of the coding sequences in each exon, as well as those of the *SPINK1* genomic sequence cloned into the pcDNA3.1/V5-His-TOPO vector, are indicated in accordance with NM_001379610. **b** Illustration demonstrating how the FLGSA assay was integrated with SpliceAI to prospectively evaluate the splicing effects of all potential coding variants within the *SPINK1* gene. *Abbreviations:* FLGSA, full-length gene splicing assay; RT-PCR, reverse transcription-PCR; SNVs, single-nucleotide variants

The primary objective of this study was to prospectively interpret the splicing impact of all potential coding SNVs within the *SPINK1* gene by leveraging a synergistic combination of the FLGSA assay and SpliceAI predictions. Our hypothesis was grounded in the belief that insights derived from correlating experimental data obtained through FLGSA with SpliceAI predictions for a subset of *SPINK1* coding SNVs could be reasonably extrapolated to the broader pool of unanalyzed *SPINK1* coding SNVs. The study would begin with a retrospective correlation analysis (using previously FLGSA-analyzed *SPINK1* coding SNVs), advance to a prospective correlation analysis (involving newly FLGSA-tested *SPINK1* coding SNVs), followed by data extrapolation, and end with further validation (Fig. 1).

### SpliceAI

SpliceAI provides four Δ scores: acceptor gain (AG), acceptor loss (AL), donor gain (DG), and donor loss (DL). These scores represent the maximum difference between the probability of the variant and the reference alleles concerning splice-altering. The Δ score ranges from 0 to 1, with higher scores indicating a greater likelihood that the variant affects splicing. Variants with a Δ score of < 0.20 were generally considered unlikely to have a substantial impact on splicing, while variants with a Δ score exceeding 0.80 were generally associated with a high specificity for splicing alterations [12]. SpliceAI also provides the pre-mRNA positions of the predicted splicing effect with respect to the variant position. For *SPINK1* variants, positive and negative pre-mRNA positions indicate positions 5’ and 3’ to the variant position in terms of the gene’s sense strand.

Our retrospective analysis involved comparing FLGSA data with SpliceAI predictions for known *SPINK1* coding SNVs. For our prospective analysis, we selected new *SPINK1* coding SNVs for FLGSA analysis based on SpliceAI-predicted Δ scores. These steps relied on SpliceAI Δ scores obtained from using the default settings of SpliceAI in February 2020. These SpliceAI Δ scores correspond to Illumina’s precomputed scores [46] created using Gencode v24 and max distance = 50 bp at the time [12] and align with those accessible to academic users on the SpliceAI-visual website [47]. Importantly, in May 2023, SpliceAI retired these Illumina precomputed scores. To adapt to this change and refine the cross-correlation, we additionally conducted a second-step analysis for *SPINK1* coding SNVs that underwent the FLGSA assay, utilizing SpliceAI Δ scores obtained from SpliceAI Lookup [48] with the following parameters: (i) Genome version, hg38; (ii) Score type, Raw; and (iii) Max distance, 10,000. This new set of SpliceAI Δ scores was manually obtained in October 2023.

### Collation of known *SPINK1* coding variants with FLGSA data

To date, the FLGSA assay has been employed to analyze 27 clinically identified *SPINK1* coding SNVs, comprising 24 missense variants and 3 synonymous variants [8, 38]. All these 27 variants were included in our retrospective correlation analysis.

### Selection of potential *SPINK1* coding variants for FLGSA

We conducted a rigorous selection process to identify potential *SPINK1* coding variants for FLGSA. This process involved a comprehensive assessment of Illumina’s precomputed SpliceAI Δ scores for all 720 potential coding SNVs, resulting from the multiplication of 240 coding nucleotides by 3, within the *SPINK1* gene. Our selection was methodically carried out for each of the gene's four exons. Typically, we included all three potential SNVs at both the start and end of each exon, with the exception of the start of exon 1 and the end of exon 4, regardless of their SpliceAI Δ scores. We gave priority to SNVs with at least one SpliceAI Δ score ≥ 0.20. Nonetheless, we excluded Δ scores ≥ 0.20 deemed physiologically irrelevant, such as high DL scores indicating non-existent splice donor sites in exon 1. Additionally, we deliberately incorporated certain variants, usually affecting the same nucleotide as a variant with a high Δ score, predicted to have no impact on splicing, into the FLGSA assay. This initial selection resulted in 35 SNVs. For further validation purposes, an additional five SNVs were chosen for FLGSA analysis. More detailed information is provided in the *Results* section.

### FLGSA

The newly selected *SPINK1* coding SNVs underwent FLGSA analysis, as previously described [34, 36, 39]. Specifically, the introduction of the selected variants into the full-length gene expression vector containing the WT *SPINK1* genomic sequence [33] and the subsequent confirmation of the introduced variants through Sanger sequencing were executed by GENEWIZ Biotech Co. (Suzhou, China). All subsequent experimental procedures were conducted at the Shanghai Changhai laboratory.

#### Cell culture, transfection, RNA extraction, and reverse transcription (RT)

Human embryonic kidney 293 T (HEK293T) cells were cultured in the DMEM basic medium (Gibco) with 10% fetal calf serum (Procell). 3.5 × 10^5^ cells were seeded per well in 6-well plates 24 h before transfection. 2.5 µg of either WT or variant plasmid, mixed with HieffTrans Universal Transfection Reagent (Yeasen), was used for transfection per well. Forty-eight hours after transfection, total RNA was extracted using the FastPure Cell/Tissue Total RNA Isolation Kit V2 (+ gDNA wiper) (Vazyme). RT was carried out using the HiScript III 1st Strand cDNA Synthesis Kit (Vazyme), incorporating 2 µL of 5 × gDNA wiper Mix, 2 µL of 10 × RT Mix, 2 µL of HiScript III Enzyme Mix, 1 µL of Oligo (dT)20VN, and 1 µg of total RNA.

#### RT-PCR and sequencing of the resulting products

RT-PCR was performed in a 25-μL reaction mixture containing 12.5 μL 2 × Taq Master Mix (Vazyme), 1 μL cDNA, and 0.4 μM of each primer. The primers used were 5’-GGAGACCCAAGCTGGCTAGT-3’ (forward) and 5’-AGACCGAGGAGAGGGTTAGG-3’ (reverse), both of which are located within the pcDNA3.1/V5-His-TOPO vector sequence. The PCR program had an initial denaturation step at 94 °C for 5 min, followed by 35 cycles of denaturation at 94 °C for 30 s, annealing at 55 °C for 30 s, and extension at 72 °C for 5 min, and a final extension step at 72 °C for 7 min. RT-PCR products presenting either a single band or multiple bands were excised from the agarose gel and then purified using a Gel Extraction Kit (Omega Bio-Tek). The sequencing primers employed were identical to those used for the RT-PCR analyses. Sequencing reactions were conducted using the BigDye Terminator v3.1 Cycle Sequencing Kit (Applied Biosystems).

#### Approximate estimate of relative expression levels of co-expressed WT and aberrant transcripts

To estimate the relative expression levels of aberrantly spliced transcripts in comparison to normally spliced transcripts for variants that produced both types of transcripts, we employed ImageJ software [49] for quantifying the relative intensities of corresponding RT-PCR bands.

### The contribution of generative artificial intelligence to the writing process

We used ChatGPT-4 [50] to enhance the readability and linguistic quality of this manuscript. We take full responsibility for the content presented herein.

## Results

### Illumina precomputed SpliceAI Δ scores and graphical illustrations

*SPINK1* is located on the reverse strand of chromosome 5. The precomputed SpliceAI Δ scores for each potential SNV at every coding site within the *SPINK1* gene were initially aligned with the hg19(chr5) coordinates [46]. These scores have been recalibrated to reflect the coding sequence of *SPINK1* from the 5' to 3' end and are detailed in Additional file 1. Specifically, the coding nucleotides of exon 1 (c.1_55), exon 2 (c.56_87), exon 3 (c.88_194), and exon 4 (c.195_240) correspond to the reverse complements of the hg19(chr5) coordinates at positions 147,211,086–147,211,140, 147,209,162–147,209,193, 147,207,585–147,207,691, and 147,204,224–147,204,269, respectively.

**Fig. 2:**
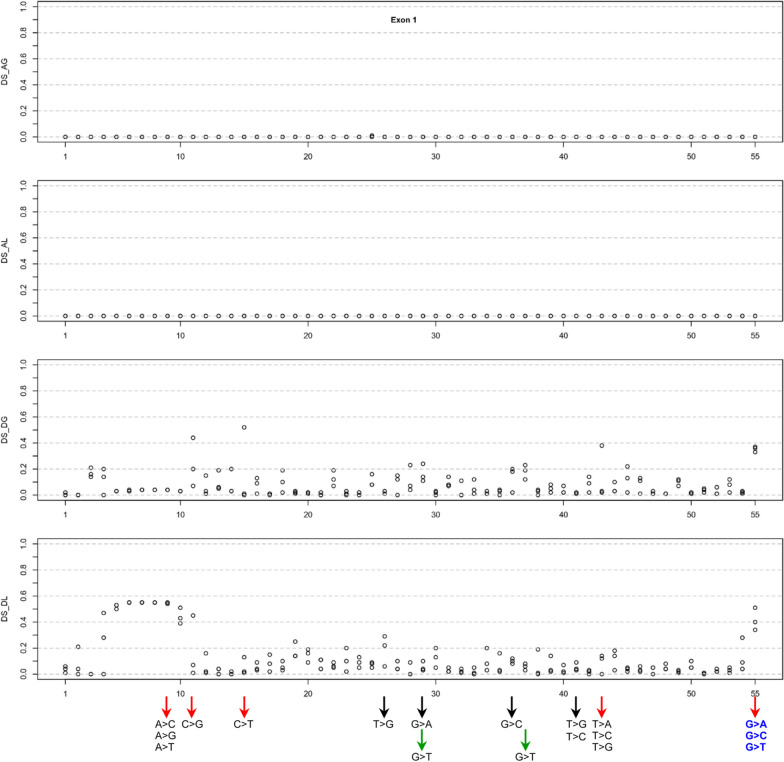
Graphical illustration of the SpliceAI Δ scores for three potential single-nucleotide variants at each coding position within *SPINK1* exon 1. The x-axis enumerates the coding positions to correlate Δ scores with specific nucleotide changes. Variants subjected to full-length gene splicing assay are highlighted at the figure's bottom, with arrows denoting their analysis status: black for previously analyzed variants, red for those currently assessed in the initial step of prospective analysis, and green for variants in the further validation phase. Variant labels are styled to indicate transcript outcomes: variants producing solely normally spliced transcripts are in standard font, while those resulting in both normally spliced and aberrantly spliced transcripts are highlighted in bold blue. *Abbreviations:* DS, Δ score; AG, acceptor gain; AL, acceptor loss; DG, donor gain; DL, donor loss

**Fig. 3:**
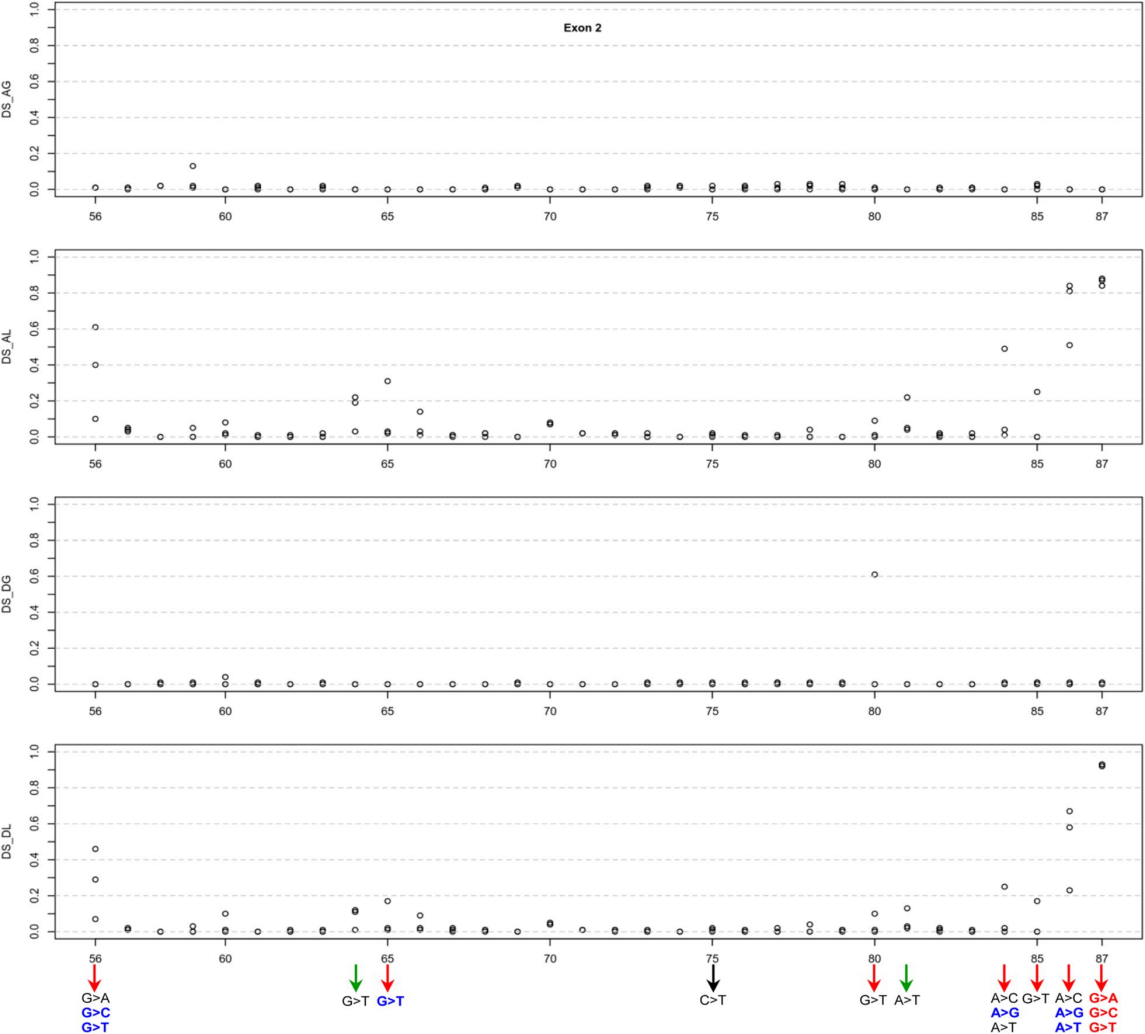
Graphical illustration of the SpliceAI Δ scores for three potential single-nucleotide variants at each coding position within *SPINK1* exon 2. The x-axis enumerates the coding positions to correlate Δ scores with specific nucleotide changes. Variants subjected to full-length gene splicing assay are highlighted at the figure's bottom, with arrows denoting their analysis status: black for previously analyzed variants, red for those currently assessed in the initial step of prospective analysis, and green for variants in the subsequent validation phase. The typographic treatment of variant names reflects their transcript profiles: standard font denotes variants leading to only normally spliced transcripts, bold blue highlights variants associated with both normally spliced and aberrantly spliced transcripts, and bold red identifies variants exclusively resulting in aberrantly spliced transcripts. *Abbreviations:* DS, Δ score; AG, acceptor gain; AL, acceptor loss; DG, donor gain; DL, donor loss

**Fig. 4:**
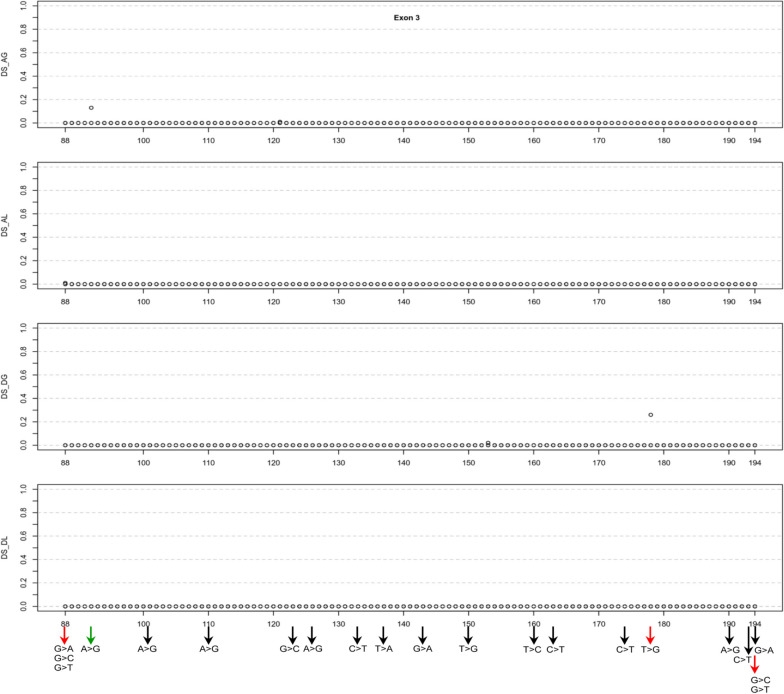
Graphical illustration of the SpliceAI Δ scores for three potential single-nucleotide variants at each coding position within *SPINK1* exon 3. The x-axis enumerates the coding positions to correlate Δ scores with specific nucleotide changes. Variants subjected to full-length gene splicing assay are highlighted at the figure's bottom, with arrows denoting their analysis status: black for previously analyzed variants, red for those currently assessed in the initial step of prospective analysis, and green for variants in the subsequent validation phase. All variants generated exclusively normally spliced transcripts. *Abbreviations:* DS, Δ score; AG, acceptor gain; AL, acceptor loss; DG, donor gain; DL, donor loss

**Fig. 5:**
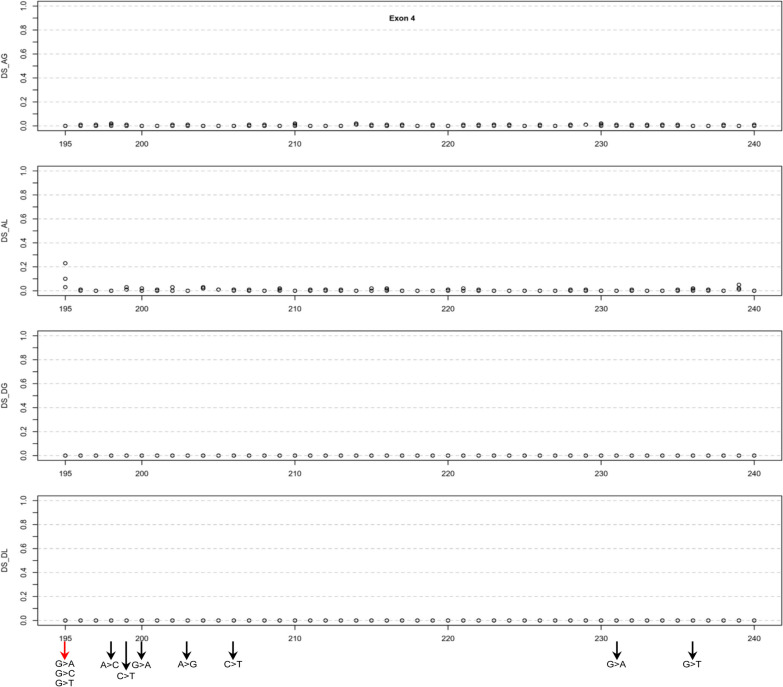
Graphical illustration of the SpliceAI Δ scores for three potential single-nucleotide variants at each coding position within *SPINK1* exon 4. The x-axis enumerates the coding positions to correlate Δ scores with specific nucleotide changes. Variants subjected to full-length gene splicing assay are highlighted at the figure's bottom, with arrows denoting their analysis status: black for previously analyzed variants and red for those currently assessed in the initial step of prospective analysis. All variants generated exclusively normally spliced transcripts. *Abbreviations:* DS, Δ score; AG, acceptor gain; AL, acceptor loss; DG, donor gain; DL, donor loss

Graphical representations have been created to elucidate the AG, AL, DG, and DL scores for the three potential SNVs at each coding site, along with the variants analyzed via FLGSA. These visual aids are presented in the context of each *SPINK1* exon (Figs. 2–5) to enhance understanding and facilitate discussion of the findings in subsequent sections.

### Retrospective correlation of FLGSA data with SpliceAI predictions for known *SPINK1* coding SNVs

We initiated the study with a retrospective analysis involving known *SPINK1* coding SNVs that had previously undergone FLGSA analysis. All 27 such variants (5 in exon 1, 1 in exon 2, 14 in exon 3, and 7 in exon 4) consistently yielded WT transcripts in the FLGSA assay [8, 38]. Details of these variants, including their precomputed SpliceAI Δ scores by Illumina [46], are provided in Table 1.

**Table 1:** FLGSA data and Illumina precomputed SpliceAI Δ scores for 27 known and 35 newly selected potential *SPINK1* coding SNVs

Exon	Variant^a^		Illumina precomputed SpliceAI scores^b^				Generation of aberrantly spliced transcripts as determined by FLGSA^c^	Study
	Nucleotide change	Amino acid change	AG	AL	DG	DL		
1	c.9A > C	p.Val3Val	0.00	0.00	0.04	0.55 (3 bp)^d^	No	This study
1	c.9A > G	p.Val3Val	0.00	0.00	0.04	0.54 (3 bp)^d^	No	This study
1	c.9A > T	p.Val3Val	0.00	0.00	0.04	0.55 (3 bp)^d^	No	This study
1	c.11C > G	p.Thr4Arg	0.00	0.00	0.44 (5 bp)	0.45 (-44 bp)	No	This study
1	c.15C > T	p.Gly5Gly	0.00	0.00	0.52 (2 bp)	0.13 (-40 bp)	No	This study
1	c.26 T > G	p.Leu9Arg	0.00	0.00	0.03	0.29 (20 bp)^d^	No	[38]
1	c.29G > A	p.Ser10Asn	0.00	0.00	0.11 (23 bp)	0.03	No	[38]
1	c.36G > C	p.Leu12Phe	0.00	0.00	0.02	0.10 (30 bp)^d^	No	[38]
1	c.41 T > C	p.Leu14Pro	0.00	0.00	0.01	0.04	No	[38]
1	c.41 T > G	p.Leu14Arg	0.00	0.00	0.02	0.09 (35 bp)^d^	No	[38]
1	c.43 T > A	p.Leu15Met	0.00	0.00	0.02	0.12 (37 bp)^d^	No	This study
1	c.43 T > C	p.Leu15 =	0.00	0.00	0.03	0.14 (37 bp)^d^	No	This study
1	c.43 T > G	p.Leu15Val	0.00	0.00	0.38 (1 bp)	0.00	No	This study
1	c.55G > A	p.Gly19Ser	0.00	0.00	0.36 (49 bp)	0.40 (0 bp)	Yes (Intron 1 retention^e^/normally spliced: 1/9.03)	This study
1	c.55G > C	p.Gly19Arg	0.00	0.00	0.33 (49 bp)	0.34 (0 bp)	Yes (Intron 1 retention^e^/normally spliced: 1/21.72)	This study
1	c.55G > T	p.Gly19Cys	0.00	0.00	0.37 (49 bp)	0.51 (0 bp)	Yes (Intron 1 retention^e^/normally spliced: 1/9.38)	This study
2	c.56G > A	p.Gly19Asp	0.01	0.10 (0 bp)	0.00	0.07	No	This study
2	c.56G > C	p.Gly19Ala	0.01	0.40 (0 bp)	0.00	0.29 (−31 bp)	Yes (E2 skipping/normally spliced: 1/1.32)	This study
2	c.56G > T	p.Gly19Val	0.01	0.61 (0 bp)	0.00	0.46 (−31 bp)	Yes (E2 skipping/normally spliced: 2.97/1)	This study
2	c.65G > T	p.Gly22Val	0.00	0.31 (9 bp)	0.00	0.17 (−22 bp)	Yes (E2 skipping/normally spliced: 1/5.16)	This study
2	c.75C > T	p.Ser25 =	0.00	0.02	0.00	0.02	No	[38]
2	c.80G > T	p.Gly27Val	0.00	0.09	0.61 (2 bp)	0.10 (−7 bp)	No	This study
2	c.84A > C	p.Arg28Ser	0.00	0.01	0.00	0.00	No	This study
2	c.84A > G	p.Arg28 =	0.00	0.49 (28 bp)	0.00	0.25 (−3 bp)	Yes (E2 skipping/normally spliced: 10.80/1)	This study
2	c.84A > T	p.Arg28Ser	0.00	0.04	0.01	0.02	No	This study
2	c.85G > T	p.Glu29*	0.00	0.25 (29 bp)	0.00	0.17 (−2 bp)	No	This study
2	c.86A > C	p.Glu29Ala	0.00	0.51 (30 bp)	0.01	0.23 (−1 bp)	No	This study
2	c.86A > G	p.Glu29Gly	0.00	0.84 (30 bp)	0.00	0.67 (−1 bp)	Yes (E2 skipping/normally spliced: 4.13/1)	This study
2	c.86A > T	p.Glu29Val	0.00	0.81 (30 bp)	0.00	0.58 (−1 bp)	Yes (E2 skipping/normally spliced: 1/5.31)	This study
2	c.87G > A	p.Glu29 =	0.00	0.87 (31 bp)	0.00	0.92 (0 bp)	Yes (Complete E2 skipping)	This study
2	c.87G > C	p.Glu29Asp	0.00	0.84 (31 bp)	0.01	0.93 (0 bp)	Yes (Complete E2 skipping)	This study
2	c.87G > T	p.Glu29Asp	0.00	0.88 (31 bp)	0.00	0.93 (0 bp)	Yes (Complete E2 skipping)	This study
3	c.88G > A	p.Ala30Thr	0.00	0.00	0.00	0.00	No	This study
3	c.88G > C	p.Ala30Pro	0.00	0.00	0.00	0.00	No	This study
3	c.88G > T	p.Ala30Ser	0.00	0.01	0.00	0.00	No	This study
3	c.101A > G	p.Asn34Ser	0.00	0.00	0.00	0.00	No	[38]
3	c.110A > G	p.Asn37Ser	0.00	0.00	0.00	0.00	No	[38]
3	c.123G > C	p.Lys41Asn	0.00	0.00	0.00	0.00	No	[38]
3	c.126A > G	p.Ile42Met	0.00	0.00	0.00	0.00	No	[38]
3	c.133C > T	p.Pro45Ser	0.00	0.00	0.00	0.00	No	[38]
3	c.137 T > A	p.Val46Asp	0.00	0.00	0.00	0.00	No	[38]
3	c.143G > A	p.Gly48Glu	0.00	0.00	0.00	0.00	No	[38]
3	c.150 T > G	p.Asp50Glu	0.00	0.00	0.00	0.00	No	[38]
3	c.160 T > C	p.Tyr54His	0.00	0.00	0.00	0.00	No	[38]
3	c.163C > T	p.Pro55Ser	0.00	0.00	0.00	0.00	No	[38]
3	c.174C > T	p.Cys58 =	0.00	0.00	0.00	0.00	No	[38]
3	c.178 T > G	p.Leu60Val	0.00	0.00	0.26 (1 bp)	0.00	No	This study
3	c.190A > G	p.Asn64Asp	0.00	0.00	0.00	0.00	No	[38]
3	c.193C > T	p.Arg65Trp	0.00	0.00	0.00	0.00	No	[38]
3	c.194G > A	p.Arg65Gln	0.00	0.00	0.00	0.00	No	[8]
3	c.194G > C	p.Arg65Pro	0.00	0.00	0.00	0.00	No	This study
3	c.194G > T	p.Arg65Leu	0.00	0.00	0.00	0.00	No	This study
4	c.195G > A	p.Arg65 =	0.00	0.03	0.00	0.00	No	This study
4	c.195G > C	p.Arg65 =	0.00	0.10 (0 bp)	0.00	0.00	No	This study
4	c.195G > T	p.Arg65 =	0.00	0.23 (0 bp)	0.00	0.00	No	This study
4	c.198A > C	p.Lys66Asn	0.01	0.00	0.00	0.00	No	[38]
4	c.199C > T	p.Arg67Cys	0.00	0.01	0.00	0.00	No	[38]
4	c.200G > A	p.Arg67His	0.00	0.00	0.00	0.00	No	[38]
4	c.203A > G	p.Gln68Arg	0.01	0.00	0.00	0.00	No	[38]
4	c.206C > T	p.Thr69Ile	0.00	0.01	0.00	0.00	No	[38]
4	c.231G > A	p.Gly77 =	0.01	0.00	0.00	0.00	No	[38]
4	c.236G > T	p.Cys79Phe	0.00	0.02	0.00	0.00	No	[38]

AG, acceptor gain; AL, acceptor loss; DG, donor gain; DL, donor loss; FLGSA, full-length gene splicing assay; SNVs, single-nucleotide variants

^a^*SPINK1* mRNA reference sequence: NM_001379610.1

^b^Information in parentheses indicates pre-mRNA positions associated with variants exhibiting a Δ score ≥ 0.10. Positive and negative positions reflect locations 5’ (upstream) and 3’ (downstream) relative to the variant, in accordance with the gene's sense strand orientation

^c^Information in parentheses indicates the ratio of aberrantly spliced to normally spliced transcripts when a variant results in both normally and aberrantly spliced transcripts. This ratio is provided as a rough estimation (refer to the main text for detailed information)

^d^Considered not to be physiologically relevant as the predicted donor loss is situated within exon 1 of the *SPINK1* gene

^e^Retention of the first 140 bases of intron 1

Among the 108 corresponding SpliceAI Δ scores, only one exceeded the threshold of 0.20. This was a DL Δ score of 0.29 (20 bp) for the variant c.26 T > G. However, it's important to note that this DL score did not have physiological relevance, as it related to the GT dinucleotide at *SPINK1* coding positions c.7_8, which is not used as a splice donor site in any of the four documented *SPINK1* transcript isoforms (NM_003122.5, NM_001354966.2, XM_047417625.1, and XM_047417626.1; with "XM" indicating predicted transcripts), as detailed in reference [51]). The next highest score was only 0.11, a DG score for the variant c.29G > A. Therefore, with the exception of the c.26 T > G variant, a perfect correlation was observed between the FLGSA-derived and SpliceAI-predicted data in the context of the subset of known *SPINK1* coding SNVs.

### Selection of potential *SPINK1* coding SNVs for FLGSA

Next, building upon Illumina’s precomputed SpliceAI scores (Additional file 1), we embarked on selecting a new cohort of *SPINK1* coding SNVs for FLGSA. Adhering to the methodological guidelines specified in the *Methods* section, we meticulously chose 35 SNVs within exon-dependent contexts.

In exon 1, which consists of 55 coding nucleotides, all 165 potential SNVs displayed AG and AL scores of zero (see Additional file 1; Fig. 2). Consequently, our selection focused on DG and DL scores. Initially, we included the three possible SNVs at the terminal position of exon 1 (c.55G > A, c.55G > C, and c.55G > T), with DG and DL scores ranging from 0.33 to 0.51. Subsequently, from the remaining SNVs, we selected the three with the highest DG scores (c.11C > G, 0.44; c.15C > T, 0.52; and c.43 T > G, 0.38), and added two more variants at c.43 (c.43 T > C and c.43 T > A) for further analysis. Regarding DL scores, we considered those with positive position values, which indicate potential loss of donor splice sites within the 5’-untranslated region or the coding sequence of exon 1, and certain negative position values, also suggesting donor splice site loss within the coding sequence of exon 1, as physiologically irrelevant. These included the cluster of high DL scores associated with variants spanning positions c.4_11. Importantly, all five variants with a physiologically relevant DL score above 0.10 (c.11C > G at 0.45; c.15C > T at 0.13; c.55G > A at 0.40; c.55G > C at 0.34; and c.55G > T at 0.51) also had a DG score of at least 0.33 and were thus already included in our selection. For comparative purposes, we also included all three SNVs at c.9, each predicted to have a DG score of 0.04.

Exon 2, encompassing 32 coding nucleotides, hosts 96 potential SNVs. Notably, two SNVs at the starting position (c.56) and all six SNVs at the final two positions (c.86 and c.87) of exon 2 demonstrated AL and DL scores > 0.20 (Additional file 1; Fig. 3). As a result, we included all potential SNVs at these three positions in the functional analysis. Additionally, the sole additional SNV meeting the criteria of both AL and DL scores > 0.20 was c.84A > G. Hence, we incorporated this SNV, along with the other two possible SNVs at the c.84 position, into the functional analysis. Finally, four variants demonstrated a single score surpassing 0.20. These included c.64G > T with an AL score of 0.22, c.65G > T with an AL score of 0.31, c.80G > T with a DG score of 0.61, and c.85G > T with an AL score of 0.25. For the FLGSA assay, we selected the latter three variants for inclusion.

Exon 3, comprising 107 coding nucleotides, contains 321 potential SNVs. Among the 1284 SpliceAI scores associated with these variants, most were zero (Additional file 1; Fig. 4). However, exceptions included an AG score of 0.13 for c.92A > G and a DG score of 0.26 for c.178 T > G. In our FLGSA analysis, we prioritized c.178 T > G. Additionally, we incorporated three SNVs at the beginning of exon 3 (c.88G > A, c.88G > C, and c.88G > T) and two at its end (c.194G > C and c.194G > T, with the note that c.194G > A had been previously analyzed in [8]).

Exon 4, with 46 coding nucleotides, contains 138 possible SNVs. All 552 corresponding SpliceAI Δ scores consistently remained at or near zero, with a maximum of 0.05, except for two cases: an AL score of 0.10 for c.195G > C and an AL score of 0.23 for c.195G > T (Additional file 1; Fig. 5). Since c.195 is the starting position of exon 4, we included all three possible SNVs at this position for FLGSA.

Details of the 35 chosen SNVs, along with their corresponding precomputed SpliceAI scores, are provided in Table 1. Additionally, the rationale behind their selection is briefly summarized in Additional file 1.

### FLGSA assay for the 35 prospectively selected *SPINK1* coding SNVs

**Fig. 6:**
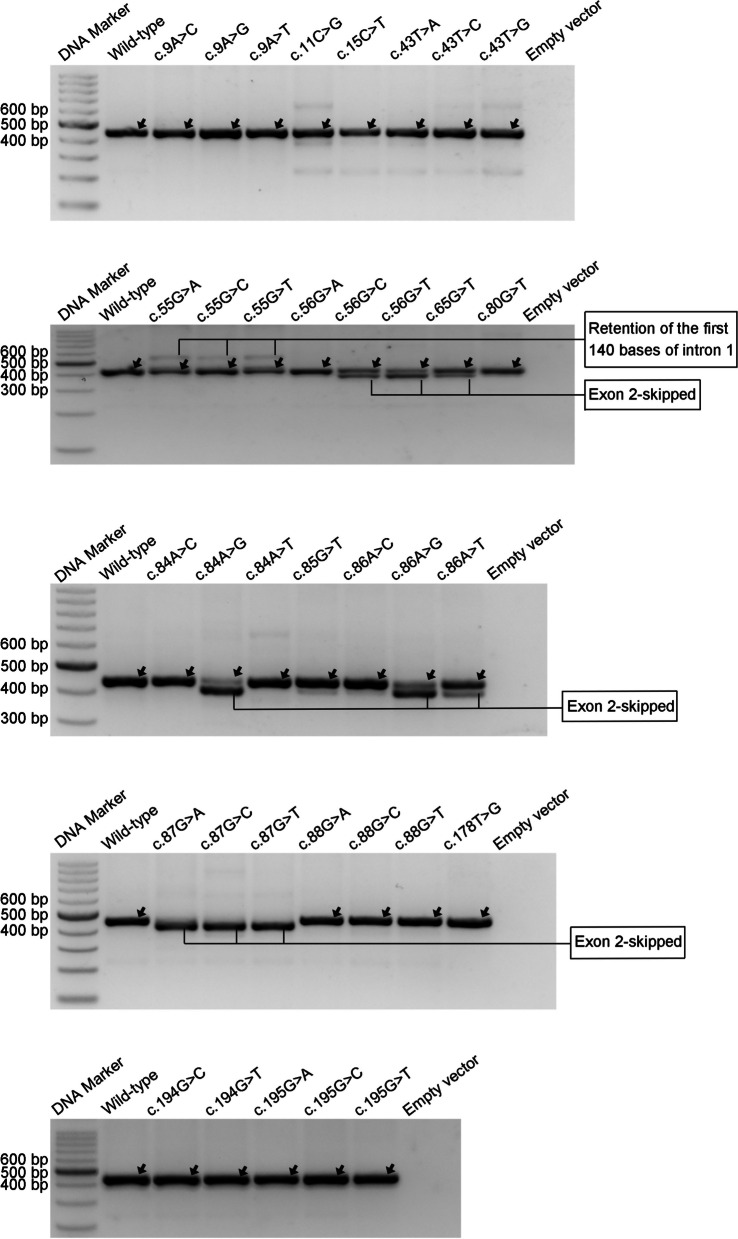
RT-PCR results from the FLGSA analysis of 35 potential *SPINK1* coding variants. Each band that underwent successful Sanger sequencing has been systematically annotated. These bands have been classified as either normally spliced transcripts (indicated by arrows) or aberrantly spliced transcripts, characterized by the retention of the first 140 bases of intron 1 or the skipping of exon 2. It is noteworthy that in cases where a variant produced two successfully sequenced bands, certain bands may contain both normally spliced and aberrantly spliced transcript isoforms. For further details and interpretation of these findings, refer to the main text. Full-length, unaltered gel images corresponding to this figure are made accessible in Additional file 2. A*bbreviations:* FLGSA, full-length gene splicing assay. RT-PCR, reverse transcription-PCR

Then, we advanced to functionally characterize the splicing effects of the 35 prospectively selected *SPINK1* coding SNVs using the FLGSA assay. The results, represented by RT-PCR band patterns in agarose gel analysis, are shown in Fig. 6. In accordance with our common practice [34, 36, 37], we employed Sanger sequencing to determine the identity of RT-PCR bands whenever possible. This step carried particular significance for two primary reasons: (i) a seemingly normally spliced RT-PCR band could differ from the genuine WT by only one or two base pairs [37, 39], and (ii) this information was pivotal for comparison with SpliceAI-predicted splice-altering sites in instances of aberrant splicing. Additionally, it's worth mentioning that the normally spliced transcripts originating from cells transfected with the variant expression vectors consistently contained the corresponding coding SNVs.

We attempted but failed to obtain readable results from Sanger sequencing for some faint RT-PCR bands, such as the one beneath the major band in the c.11C > G variant (Fig. 6). These faint bands may signify authentic aberrantly spliced transcripts; however, their extremely low intensity compared to normal transcripts implies a lack of clinical relevance. This is based on the rationale that a *SPINK1* variant causing < 10% functional loss (or retaining > 90% function) is unlikely to be pathologically significant [32]. Therefore, these faint bands were not further pursued in our analysis.

RT-PCR bands that were successfully Sanger sequenced are highlighted in Fig. 6. Among the 35 variants, 23 yielded exclusively normally spliced transcripts, while 12 resulted in aberrant splicing. Of these 12, three variants at the terminal position of exon 3 (c.87G > A/C/T) exclusively produced exon 2-skipped transcripts. Six variants in exon 2 (c.56G > C/T, c.65G > T, c.84A > G, c.86A > G/T) yielded a mix of normal and exon 2-skipped aberrant transcripts. The three variants at the terminal position of exon 1 (c.55G > A/C/T) generated a mix of normally spliced and aberrantly spliced transcripts, with the latter retaining the initial 140 bp of *SPINK1* intron 1.

**Fig. 7:**
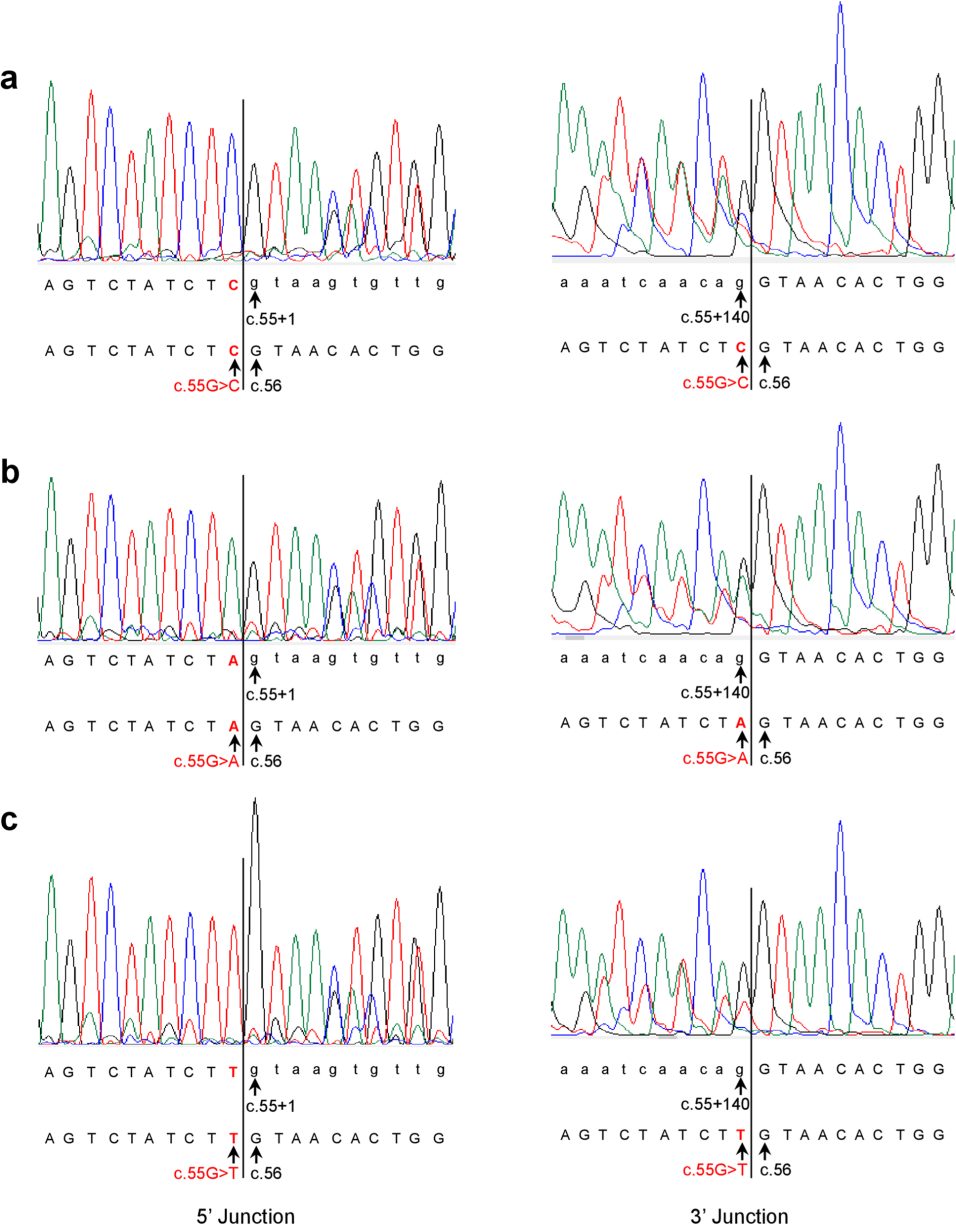
Sanger Sequencing results of 'Retention of the first 140 bases of intron 1' RT-PCR bands from three exon 1 terminal variants (c.56G > A/C/T) in *SPINK1*. Refer to Fig. 6 for the corresponding bands. Each band was found to contain a mixture of aberrantly spliced and normally spliced transcripts, with the 5’ and 3’ junctions of the aberrant transcript isoform, being delineated by vertical lines. The annotations beneath the electropherograms detail the junction-spanning sequences for both isoforms: the upper annotation for the aberrantly spliced transcript and the lower for the normally spliced transcript. In all subpanels, the normally spliced transcripts show a consistent sequence of exon 1 followed by exon 2, with the introduced exon 1 terminal variants highlighted in red. The aberrantly spliced transcripts have consistent 5’ junctions with exon 1 followed by intron 1 sequences (introduced variants in red), and their 3’ junctions are uniform, displaying retained intron 1 sequence (up to c.55 + 140) followed by exon 2. Sequence numbering is based on NM_001379610.1. Specifically, c.55 and c.56 denote the terminal position of exon 1 and the start of exon 2 in *SPINK1*, respectively; c.55 + 1 and c.55 + 140 refer to the first and the 140th nucleotides of intron 1 in *SPINK1*. *Abbreviations:* RT-PCR, reverse transcription-PCR

**Fig. 8:**
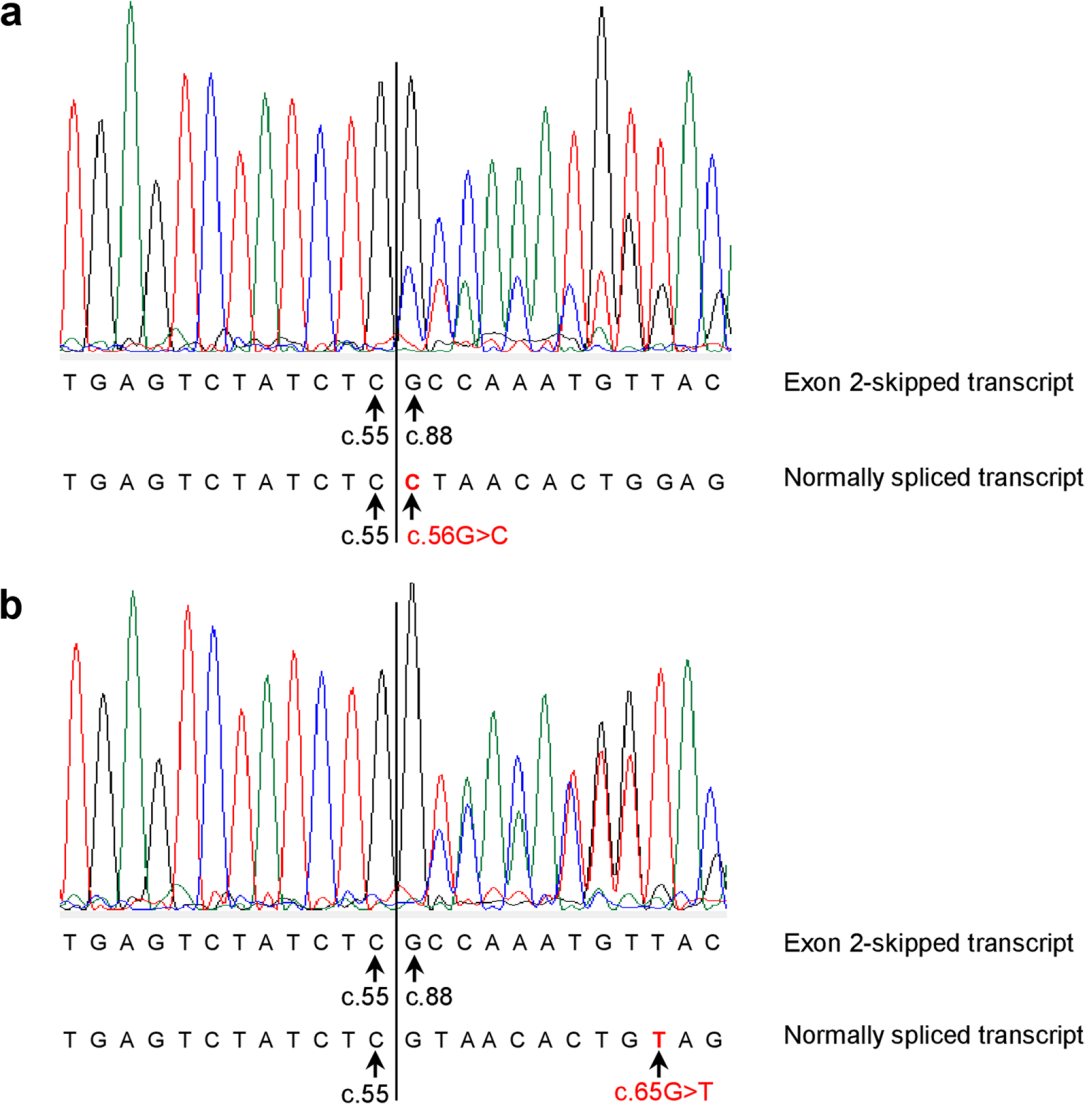
Sanger sequencing of 'Exon 2-skipped' RT-PCR bands from two exon 2 variants (c.56G > C and c.65G > T) in *SPINK1*. For the corresponding bands, refer to Fig. 6. Sanger sequencing revealed that each band contains a mix of aberrantly spliced and normally spliced transcripts, with the junctions of both transcript isoforms being delineated by vertical lines. Annotations beneath the electropherograms specify the junction-spanning sequences for both isoforms: the upper annotation pertains to the aberrantly spliced transcript, and the lower to the normally spliced transcript. In both panels, the normally spliced transcripts exhibit a consistent sequence of exon 1 followed by exon 2, differentiated only by the introduced variants (highlighted in red). The aberrantly spliced transcripts are identical, characterized by exon 1 directly followed by exon 3. Sequence numbering aligns with NM_001379610.1. Specifically, c.55, c.56, and c.88 mark the terminal position of exon 1, the start of exon 2, and the start of exon 3 in *SPINK1*, respectively

It is imperative to highlight that in cases where variants resulted in two successfully sequenced RT-PCR bands, some bands contained both normally spliced and aberrantly spliced transcript isoforms. This occurrence, likely due to hybrid dimer formation among co-existing isoforms under our experimental conditions, is exemplified by the sequencing of 'aberrant RT-PCR bands' derived from five different variants. Specifically, Fig. 7 illustrates the sequencing of the weaker upper 'Retention of the first 140 bases of intron 1' RT-PCR bands from three exon 1 terminal variants (c.55G > C/T/A), which include normally spliced transcripts. Similarly, Fig. 8 provides details on the sequencing of the weaker lower 'Exon 2-skipped' RT-PCR bands from two exon 2 variants (c.56G > C and c.65G > T), also containing normally spliced transcripts. These two figures serve not only to reveal the identities of the two distinct aberrant transcripts but also the identities of the introduced exonic variants.

To estimate the relative expression levels of normally and aberrantly spliced transcripts for the nine variants producing two distinct, successfully sequenced RT-PCR bands, we utilized ImageJ for band quantification. The outcomes of this analysis, along with the primary FLGSA results, are provided in Table 1. It's crucial to note that this quantification directly contrasted the bands for normally spliced and aberrantly spliced transcripts as depicted in Fig. 6, without accounting for the presence of both transcript isoforms within some bands. Essentially, this represents an approximate analysis and did not alter our main findings.

### Correlation of FLGSA data with SpliceAI predictions for the 35 prospectively analyzed SNVs

Subsequently, we conducted a thorough analysis to correlate the FLGSA data generated for the prospectively examined 35 SNVs with their corresponding Illumina precomputed SpliceAI scores (Table 1). After discarding physiologically irrelevant DL scores linked to various SNVs in exon 1, we observed a significant pattern in relation to the threshold Δ scores of 0.20 and 0.80 [12]. Specifically, all variants with a Δ score not exceeding 0.20 exclusively produced normally spliced transcripts. Conversely, every variant with a Δ score above 0.80 consistently led to the production of aberrant variants.

Establishing a clear correlation between the presence or absence of aberrant transcripts and intermediate Δ scores (0.20 to 0.80) was challenging. However, a notable pattern emerged upon analyzing Δ scores for SNVs at positions c.55, c.56, and c.86. Each of these positions underwent FLGSA analysis, with at least two of the three possible SNVs at each position generating both aberrant and normal transcripts. For a more focused analysis, we will compare the DL scores associated with these SNVs. At position c.55, all three SNVs yielded both aberrantly and normally spliced transcripts (Table 1). Notably, the variant with the lowest DL score, c.55G > C (0.34), also had the lowest ratio of aberrantly to normally spliced transcripts. At c.56, the c.56G > A variant, with no scores above 0.20, produced only normally spliced transcripts. In contrast, c.56G > C and c.56G > T, with DL scores of 0.29 and 0.46 respectively, yielded both transcript types, and their aberrant/normal transcript ratios aligned with their DL scores. Regarding c.86, c.86A > C, which had the lowest DL score (0.23), did not produce aberrant transcripts. Conversely, c.86A > G, with the highest DL score (0.67), resulted in a significantly higher aberrant/normal transcript ratio of 4.13/1. Interestingly, c.86A > T, with a lower DL score (0.57) than c.86A > G, led to a much lower aberrant/normal transcript ratio (1/5.31) in comparison.

We then hypothesized that conducting a comprehensive cross-comparison of various events within the same exon context might yield valuable insights into the remaining intermediate Δ scores. We explored this hypothesis within the contexts of the four exons.

#### Exon 1

**Fig. 9:**
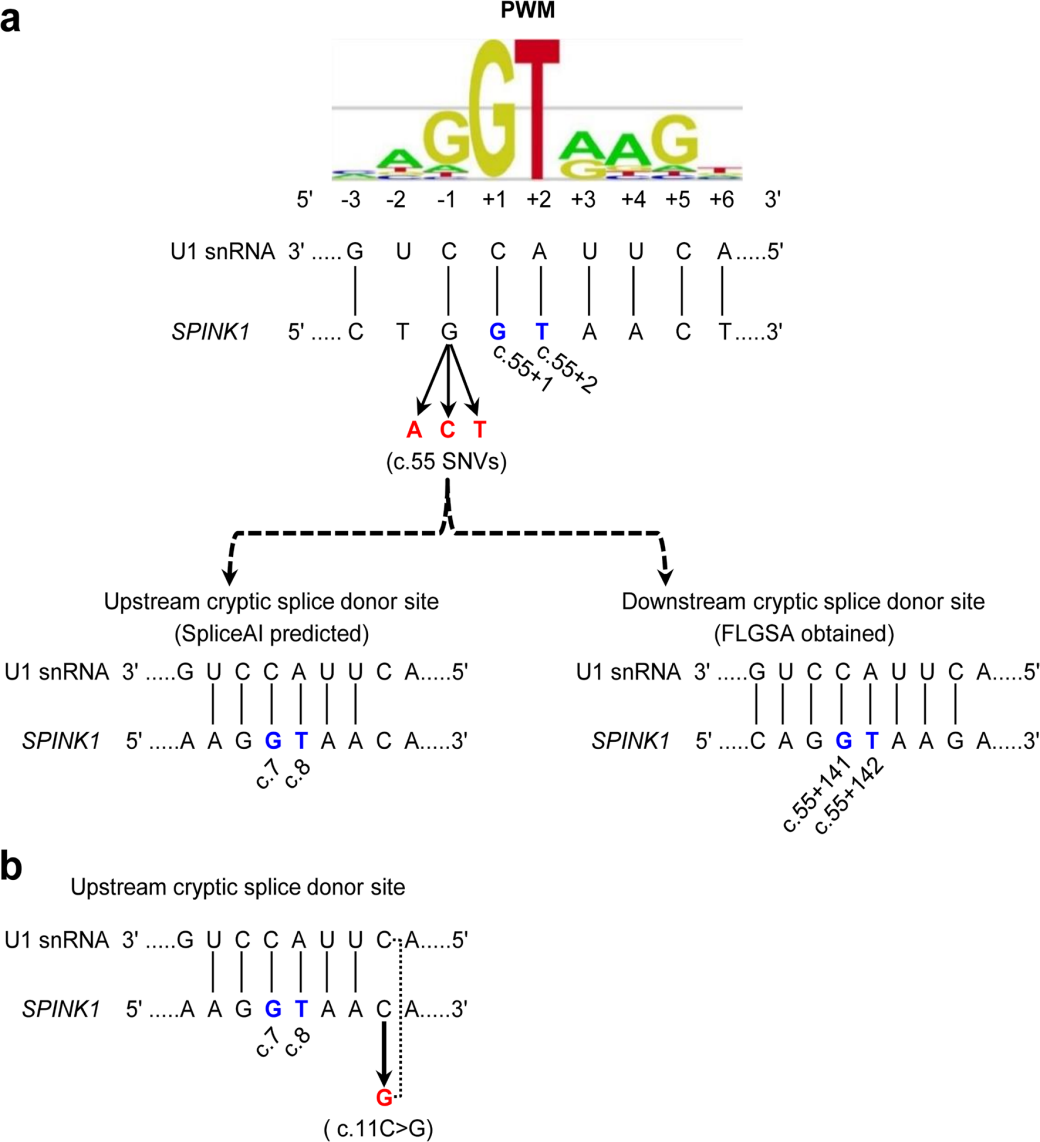
Interpretation of the three c.55 SNVs and the c.11C > G variant in exon 1 by reference to SpliceAI predictions and FLGSA results. **a** Illustration of the (partial) disruption of the physiological 5’ splice donor site of *SPINK1* intron 1 caused by the three potential SNVs at the last nucleotide of exon 1 (c.55). This disruption is shown in the context of the corresponding 9-bp 5’ splice signal sequence, which interacts with the 3’-GUCCAUUCA-5’ sequence at the 5’ end of U1snRNA. SpliceAI predicted this disruption (DL scores, 0.34 to 0.51) and the activation of an upstream cryptic splice donor site within exon 1 (DG scores, 0.33 and 0.37). However, our FLGSA assay revealed the activation of a downstream cryptic splice donor site. Vertical lines indicate paired bases between the 9-bp 5’ splice signal sequence and the 5’ end sequence of U1snRNA. The GT dinucleotides involved are highlighted in blue, with their positions (in accordance with NM_001379610.1) indicated. The 9-bp 5' splice site signal sequence position weight matrices (PWM) were sourced from Leman et al. [52], an Open Access article distributed under the terms of the Creative Commons Attribution Non-Commercial License. Note that the 9-bp 5’ splice signal sequences, whether in the context of the consensus sequence or *SPINK1* sequences, are presented in DNA. **b** Illustration of the c.11C > G variant in the context of the aforementioned upstream cryptic splice donor site. A dotted line represents the new base pairing derived from the variant, enhancing the interaction between the 9-bp 5’ splice signal sequence and the 5’ end sequence of U1snRNA

All three SNVs at the last nucleotide of exon 1, c.55, exhibited DL scores ranging from 0.34 to 0.51 and DG scores between 0.33 and 0.37 (Table 1). Based on their corresponding mRNA positions, these SNVs were predicted to disrupt the physiological GT splice donor site at positions c.55 + 1_2 and activate an upstream cryptic splice donor site within exon 1 (i.e., the GT dinucleotide at position c.7_8) (Fig. 9a). This would result in a significantly shortened transcript that lacked the last 49 nucleotides (i.e., c.7 to c.55) of exon 1. Interestingly, our FLGSA assay detected an aberrant transcript that retained the first 140 bases of intron 1 (Figs. 6 and 7), due to the activation of a downstream cryptic GT splice site located at the deep intron 1 region (precisely at c.55 + 141_142) (Fig. 9a).

We performed two additional analyses to address the discrepancy between in silico predictions and experimental data. First, considering that the Illumina precomputed scores were created using a maximum distance of 50 bp, we reevaluated the three possible c.55 SNVs using SpliceAI with an extended distance of 10,000 bp and the hg38 sequence [48]. Although the resulting DG and DL scores showed slight variations compared to the Illumina precomputed scores (Additional file 1), they did not alter the predicted splicing outcomes. Second, we examined the predicted and experimentally obtained cryptic GT donor site in the context of the 9-bp 5’ splice signal sequence and their pairing with the 3’-GUCCAUUCA-5’ sequence at the 5’ end of U1snRNA (see [39] and references therein). Interestingly, the experimentally identified cryptic GT donor site was found within a 9-bp 5’ splice signal sequence that exhibited 8 bp complementarity with the 9 bp U1snRNA sequence. In contrast, the in silico predicted cryptic GT donor site resided within a 9-bp 5’ splice signal sequence with only 6 bp complementarity to the 9 bp U1snRNA sequence (Fig. 9a). It's noteworthy that this in silico predicted cryptic GT donor site coincides with the previously discussed false physiological GT dinucleotide at position c.7_8, which was related to the DL score of the known c.26 T > G variant (see *Retrospective correlation of FLGSA data with SpliceAI predictions for known SPINK1 coding SNVs*). Based on these new findings, we speculate that SpliceAI might have favored the nearby cryptic donor site in exon 1 over the more distant cryptic donor site in intron 1.

Another variant in exon 1, c.11C > G, was predicted to induce a splicing effect similar to the three potential c.55 SNVs based on the SpliceAI scores. Interestingly, it displayed even higher DG and DL scores than those of the three potential c.55 SNVs (Table 1). However, the FLGSA analysis did not reveal aberrant transcripts associated with the c.11C > G variant. As illustrated in Fig. 9b, c.11C > G resides within the 9-bp 5’ splice signal sequence linked to the previously mentioned cryptic 5’ splice GT donor site at positions c.7_8. Notably, it increased sequence complementarity with the 9-bp U1snRNA sequence from 6 to 7 bp compared to the WT sequence. It's essential to highlight that, in this scenario, the physiological intron 1 splice donor signal sequence remains unaltered. Bearing this in mind, we conjecture that the enhanced sequence complementarity brought about by the c.11C > G variant may have encountered difficulties in competing with the intact physiological intron 1 splice donor signal sequence, which exhibited 8 bp complementarity with U1snRNA (Fig. 9a).

Shifting our focus to other variants, let's consider c.15C > T, which had the highest DG score (0.52) among all possible coding SNVs in exon 1, but it only had a DL score of 0.13. We also have c.43 T > G, which had a DG score of 0.38 but a DL score of zero. Importantly, neither of these variants resulted in the generation of aberrant transcripts (Table 1). Drawing parallels with the previously discussed c.11C > G variant, we propose that their predicted cryptic 5’ splice donor sites, located within the coding sequence of exon 1, may not have effectively competed against the intact physiological intron 1 splice donor signal sequence.

#### Exons 2–4

Moving on to exons 2–4, two variants warrant closer examination: c.80G > T in exon 2 and c.178 T > G in exon 3. The former variant displayed a DG score of 0.61 but a DL score of only 0.10, while the latter had a DG score of 0.26 but a DL score of zero (Table 1; Figs. 3–5). Our FLGSA analysis did not produce any aberrant transcripts associated with either of these variants. In line with our earlier observations concerning variants in exon 1, such as c.11C > G and c.15C > T, we propose that the predicted cryptic 5’ splice donor sites may not have effectively competed with the intact physiological intron 2 and intron 3 splice donor signal sequences, respectively.

### Extrapolation to unanalyzed *SPINK1* coding SNVs

Finally, we addressed a critical question: Can we reasonably interpret the potential splicing effects of the 658 *SPINK1* coding variants that have not yet undergone functional analysis, based on insights derived from the cross-correlation of FLGSA data and SpliceAI predictions of the 27 known and 35 newly analyzed SNVs? To accurately answer this question, we initially evaluated whether the Illumina precomputed scores significantly deviated from those calculated using a distance of 10,000 bp and the hg38 sequence. Consequently, we manually acquired these latter scores from [48] for all SNVs that underwent FLGSA. While we did observe slight disparities between the two datasets for many SNVs (see Additional file 1), it's crucial to emphasize that these variations were not expected to result in any changes to the predicted splicing outcomes. Thus, we proceeded confidently, utilizing the Illumina precomputed scores for our subsequent discussions within the contexts of the four exons. Our primary focus remained on unanalyzed SNVs with a Δ score falling within the range of 0.20 to 0.30. This choice was motivated by two factors: (i) we have already included all variants with a physiologically relevant Δ score exceeding 0.30 for FLGSA analysis, and (ii) a Δ score below 0.20 is highly unlikely to impact splicing.

#### Exon 1

In exon 1, we identified nine unanalyzed SNVs with a physiologically plausible Δ score of ≥ 0.20 (c.3G > A, c.4A > C, c.11C > T, c.14G > T, c.28A > T, c.29G > T, c.36G > A, c.37G > T, and c.45G > T). Notably, these scores consistently fall within the DG type, ranging from 0.20 to 0.24. Interestingly, all of these variants were predicted to have cryptic GT splice donor sites that coincide with the previously discussed GT at c.7_8. Additionally, these variants exhibited low DL scores, spanning from 0 to 0.12. When comparing these scores with those of the functionally analyzed exon 1 SNVs (see Table 1), we can conclude that none of these nine variants had any discernible impact on splicing.

#### Exon 2

In exon 2, we identified only two unanalyzed SNVs with a physiologically plausible Δ score of ≥ 0.20 (c.64G > T and c.81A > T). Both scores are identical (0.22) and belong to the AL type. AG and DG scores of the two variants are zero, while their DL scores are similar (0.11–0.13). Evaluation of the corresponding mRNA positions associated with the AL and DL scores demonstrated that their predicted splicing outcomes would result in exon 2 skipping.

Of the functionally analyzed exon 2 variants, c.85G > T most closely resembles c.64G > T and c.81A > T in terms of the Δ scores. However, c.85G > T had both slightly higher AL and DL scores than c.64G > T and c.81A > T (AL, 0.25 vs. 0.22; DL, 0.17 vs. 0.11–0.13) and produced no aberrant transcripts. c.65G > T is the next variant that most closely resembles c.64G > T and c.81A > T. c.65G > T had a higher AL score (0.31) but equal DL score (0.17) compared to c.85G > T and generated aberrant transcripts. However, the aberrant transcript was generated alongside the WT transcript, and its amount was much less than that of the WT transcript (ratio of 1:5.16).

Based on this cross-comparison, we can conclude that c.64G > T and c.81A > T are highly unlikely to generate aberrant transcripts.

#### Exons 3 and 4

In exon 3 and 4, none of the functionally analyzed SNVs generated aberrant transcripts (Table 1). Moreover, except for c.92A > G in exon 3, which had an AG score of 0.13, none of the unanalyzed SNVs had a SpliceAI score exceeding 0.05 (Additional file 1; Figs. 4 and 5). Consequently, all SNVs in these two exons were considered not to impact splicing.

### Further validation

**Fig. 10:**
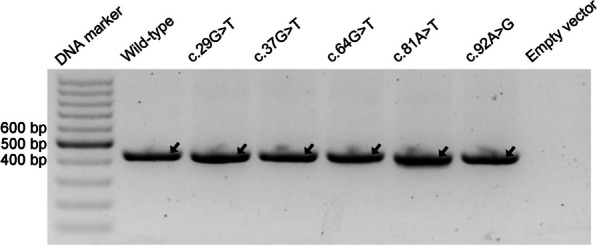
RT-PCR results for the validation analysis of five potential *SPINK1* coding variants through FLGSA. Arrows indicate wild-type or normally spliced transcripts, all of which were confirmed by Sanger sequencing. Full-length, unaltered gel image corresponding to this figure is made accessible in Additional file 3. *Abbreviations:* FLGSA, full-length gene splicing assay. RT-PCR, reverse transcription-PCR

While we had confidence in our above extrapolation, we opted for additional validation. Therefore, we selected five variants with the highest scores among those not functionally analyzed within exons 1, 2, and 3 for FLGSA analysis. Specifically, they included two of the nine exon 1 variants mentioned above (c.29G > T and c.37G > T), the two exon 2 variants mentioned earlier (c.64G > T and c.81A > T), and the exon 3 variant c.92A > G (Table 2). As shown in Fig. 10, all five variants exclusively produced WT transcripts, thereby validating our extrapolation.

**Table 2:** Selected five *SPINK1* coding SNVs for further validation*

Exon	Variant^a^		Illumina precomputed SpliceAI scores^b^			
	Nucleotide change	Amino acid change	AG	AL	DG	DL
1	c.29G > T	p.Ser10Ile	0.00	0.00	0.24 (23 bp)	0.10 (−26 bp)
1	c.37G > T	p.Ala13Ser	0.00	0.00	0.23 (31 bp)	0.08
2	c.64G > T	p.Gly22*	0.00	0.22 (8 bp)	0.00	0.11 (−23 bp)
2	c.81A > T	p.( =)	0.00	0.22 (25 bp)	0.00	0.13 (−6 bp)
3	c.92A > G	p.Lys31Arg	0.13 (−1 bp)	0.00	0.00	0.00

AG, acceptor gain; AL, acceptor loss; DG, donor gain; DL, donor loss; FLGSA, full-length gene splicing assay; SNVs, single-nucleotide variants

^*^All five variants exclusively produced normally spliced transcripts through FLGSA (see Fig. 10)

^a^*SPINK1* mRNA reference sequence: NM_001379610.1

^b^Information in parentheses indicates pre-mRNA positions associated with variants exhibiting a Δ score ≥ 0.10. Positive and negative positions reflect locations 5’ (upstream) and 3’ (downstream) relative to the variant, in accordance with the gene's sense strand orientation

### Overall correlation between FLGSA findings and SpliceAI predicted scores across all exons

**Fig. 11:**
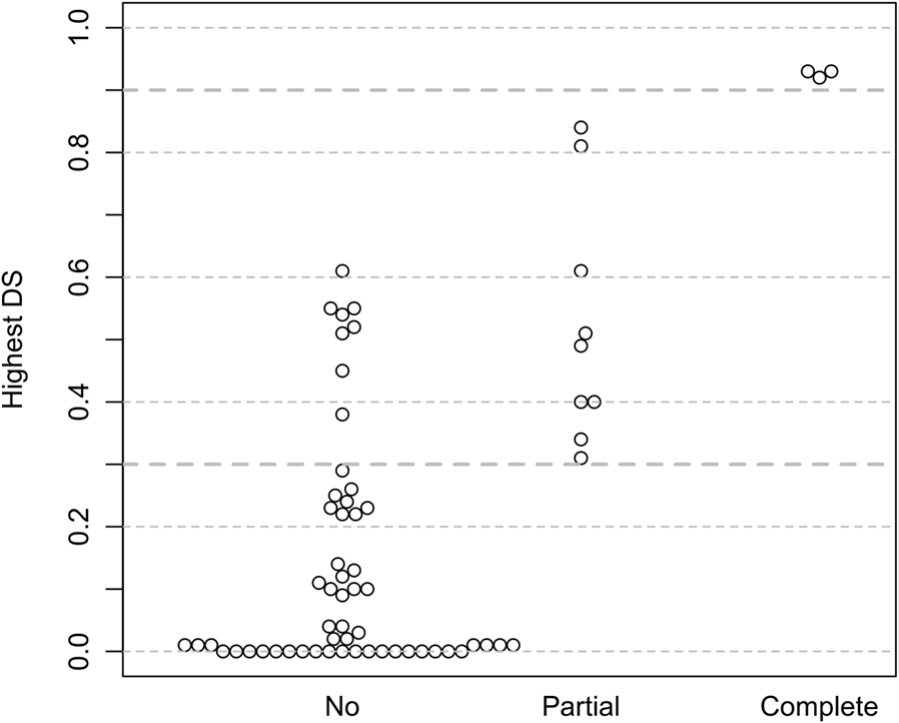
Overall correlation between FLGSA findings and SpliceAI predictions across all exons of the *SPINK1* gene. On the y-axis, "Highest DS" represents the highest Δ score among the four SpliceAI predictions for each of the 67 *SPINK1* variants analyzed through the full-length gene splicing assay (refer to Table 1 for details). The x-axis categorizes the variants based on their transcript outcomes: "No" for variants exclusively producing normally spliced transcripts, "Partial" for variants leading to a mix of normally and aberrantly spliced transcripts, and "Complete" for variants solely resulting in aberrantly spliced transcripts. Δ scores of 0.90 and 0.30 are demarcated with thicker dotted lines to denote thresholds for exclusive generation of aberrantly spliced or normally spliced transcripts, respectively

In broadening our correlation analysis beyond isolated exon assessments, we evaluated the relationship between FLGSA findings and SpliceAI predicted scores across all exons of the *SPINK1* gene. The 67 variants examined through FLGSA were categorized based on their transcript outcomes: (i) variants that exclusively produce normally spliced transcripts, (ii) variants that lead to a mixture of normally spliced and aberrantly spliced transcripts, and (iii) variants that exclusively result in aberrantly spliced transcripts. Recognizing that a single SpliceAI score might not capture the intricate nature of splicing events, we utilized the highest of the four available scores for our analysis. This approach revealed that variants with the highest SpliceAI scores > 0.80 invariably led to aberrantly spliced transcripts, with those scoring > 0.90 exclusively yielding aberrant splicing. In contrast, variants with the highest scores < 0.30 were consistently associated with the production of only normally spliced transcripts (Fig. 11).

### Overview of splice-altering coding SNVs in the *SPINK1* gene

Overall, our study covers 67 *SPINK1* coding SNVs, accounting for 9.3% of all 720 possible coding SNVs and affecting 46 (19.2%) of 240 coding nucleotides. Out of these 67 SNVs, 12 were experimentally found to impact splicing. Based on a comprehensive cross-correlation of FLGSA-obtained and SpliceAI-predicted data, we conclude that all unanalyzed potential coding SNVs in the *SPINK1* gene are unlikely to have a significant effect on splicing. Therefore, the 12 splice-altering events identified in our study represent the totality of splice-altering events among the 720 potential coding SNVs in the *SPINK1* gene. These splice-altering SNVs were found solely in exons 1 and 2, notably at the first and/or last coding nucleotides of these exons. Among the splice-altering events, 11 were missense variants, accounting for 2.17% of the 506 potential missense variants, while one was synonymous, accounting for 0.61% of the 164 potential synonymous variants (see Table 3).

**Table 3:** Summary of splice-altering coding SNVs in the *SPINK1* gene

Variant types	Total potential SNVs (a)	Splice-altering SNVs (b)	Percentage (b/a)
Translation initiation codon^a^	9	0	0
Missense	506	11	2.17
Synonymous	164	1	0.61
Nonsense	32	0	0
Translation termination codon^a^	9	0	0
Total	720	12	1.67

SNVs, single-nucleotide variants

^a^SNVs occurring within either the translation initiation or termination codon are referred to simply as "translation initiation codon" or "translation termination codon" variants

## Discussion

In this study, we leveraged the well-established FLGSA assay [8, 34–38] in conjunction with SpliceAI [12] to explore the prospective interpretation of splicing effects for all potential coding SNVs within the *SPINK1* gene, following the structured progression outlined in Fig. 1. It's pertinent to note that the field of splicing predictions has witnessed continuous improvements and advancements. Some recent tools, like SPiP [53, 54] and SpliceVault [55], claim higher accuracy than SpliceAI. Our decision to employ SpliceAI was based on its comprehensive validation and widespread acceptance as a benchmark in splicing prediction within medical genetics at the time our study commenced. While a comparison with SPiP and SpliceVault might offer additional insights, such an analysis falls outside the scope of our current research.

Our analysis revealed notable discrepancies between SpliceAI predictions and FLGSA data. Specifically, SpliceAI predicted that several exon 1 SNVs would disrupt a splice donor site within the coding sequence of exon 1 (refer to Additional file 1; Table 1). However, these predictions were contradicted by a crucial piece of evidence: none of the four documented *SPINK1* transcript isoforms [51] utilize a GT dinucleotide within the coding sequence of exon 1 as a splice donor site. This observation, coupled with our FLGSA assay results, strongly indicates that these SpliceAI predictions were inaccurate.

A further notable discrepancy involved the splicing outcomes of the three SNVs at position c.55, specifically at exon 1's terminal nucleotide. SpliceAI predicted the activation of an upstream cryptic GT dinucleotide at c.7_8, whereas our FLGSA assay identified the activation of a downstream cryptic GT dinucleotide located at c.55 + 141_142. Intriguingly, the SpliceAI-predicted cryptic GT dinucleotide coincided with one of the aforementioned erroneous splice GT donor sites, highlighting a recurring issue with SpliceAI predictions in the context of exon 1 coding sequences. Notably, our experimentally identified cryptic GT dinucleotide exhibited stronger complementarity with the 3’-GUCCAUUCA-5’ sequence at the 5’ end of U1snRNA, featuring eight complementary bases, in contrast to the SpliceAI-predicted cryptic GT dinucleotide with only six complementary bases (refer to Fig. 9a). It's important to mention that our experimentally identified cryptic GT dinucleotide is situated more distantly (141 bp) from c.55 than the SpliceAI-predicted cryptic GT dinucleotide (47 bp), potentially explaining why the latter was not correctly predicted by SpliceAI. Additionally, it's worth acknowledging that variants in exon 1 are not readily amenable to analysis through the commonly used minigene assay [9], and the activation of cryptic donor or splice sites in deep intronic regions may often elude detection via a minigene assay.

The above two notable discrepancies were consistently observed with exon 1 variants. Coincidently, the SpliceAI DG and DL scores for exon 1 variants (Fig. 2) appear considerably noisier when compared to scores for exons 2–4 (see Figs. 3–5). It is possible that these may be related to SpliceAI's training, which focuses mainly on internal exons [12]. The unique characteristics of the first exon, despite its involvement with the same core spliceosomal components as internal exons, might diminish SpliceAI's predictive accuracy for mutations there. At present, specific analytical guidelines for first or last exon variants are lacking.

Except for the aforementioned discrepancies, we found a robust correlation between FLGSA data and SpliceAI predictions, particularly concerning the 0.20 and 0.80 threshold scores and the findings for all possible SNVs at c.55, c.56, and c.86. While the relationship between FLGSA data and SpliceAI scores below 0.20 or above 0.80 tended to be straightforward, deciphering the correlation between FLGSA data and intermediate SpliceAI scores within the range of 0.20 to 0.80 presented challenges. Nevertheless, our efforts to correlate these intermediate SpliceAI scores with FLGSA findings yielded intriguing insights. For instance, variants with intermediate SpliceAI scores, when leading to aberrant transcripts, often produced a mix of aberrant and WT transcripts. Furthermore, in the case of all possible SNVs at c.55, c.56, and c.86, intermediate SpliceAI scores seemed to correlate with the aberrant/WT transcript ratio. These mutually reinforcing data were instrumental in guiding a cross-comparison concerning unanalyzed variants, allowing us to reasonably extrapolate that none of the unanalyzed coding SNVs in the *SPINK1* gene are likely to exert a significant effect on splicing.

In our study, we initially employed a 0.20 threshold score as defined in [12] for our analyses. It is crucial to recognize, however, that the applicability of this threshold score may not be uniform across different variant or gene contexts, as reported in the literature [56, 57]. This variation highlights the importance of a context-specific approach when applying such thresholds. In light of this, our extensive correlation analysis, encompassing all variants analyzed using FLGSA (illustrated in Fig. 11), revealed a noteworthy subtlety. Adjusting the cut-off to 0.30, rather than sticking with the conventional 0.20, resulted in more specific outcomes without compromising sensitivity. This adjustment indicates that even minor modifications in threshold scores can considerably refine the accuracy of our predictions, making them more appropriately tailored to the unique characteristics of the study gene. These findings underscore the significance of integrating experimental assays with in silico tools in genetic research. By doing so, we not only validate our methodologies but also pave the way for more advanced and precise analyses in future studies.

While our data, derived from the functional analysis of 67 variants and subsequent correlation with SpliceAI predictions, strongly suggest that *SPINK1* coding SNVs with a SpliceAI score below 0.20 are unlikely to significantly affect splicing, we acknowledge the theoretical possibility of exceptions. This perspective is informed by studies such as [56, 58], which demonstrate that low-scoring variants in other genes can indeed influence splicing. Given the larger number of *SPINK1* variants with scores under 0.20, a categorical dismissal of their potential impact without functional analysis may be premature. However, we assert that our extrapolation is grounded in a robust dataset, providing a reasonable basis for our conclusions while understanding the limitations inherent in any extrapolative analysis.

To the best of our knowledge, this study represents the first attempt to prospectively interpret all potential coding SNVs in a disease-associated gene. Our findings unveiled that within the *SPINK1* gene, 2.17% of all potential missense variants, 0.61% of all potential synonymous variants, but none of the potential nonsense variants have an impact on splicing. In total, 1.67% (12 out of 720) of all potential coding SNVs in the *SPINK1* gene were found to alter splicing.

Among the 12 splice-altering variants, five (c.84A > G, c.86A > G, c.87G > A, c.87G > C, and c.87G > T) led exclusively or predominantly to aberrant transcripts. These five variants can be classified as “pathogenic” and would have been mislabeled as silent or missense variants without the FLGSA assay. The remaining seven variants exhibited aberrant to WT transcript ratios ranging from 1/21.72 to 2.97/1. In all these cases, the aberrant transcripts—either retaining part of intron 1 or omitting the entire exon 2—would yield a non-functional product. However, when the aberrant transcript ratio is substantially lower than that of the WT transcript, the variant in question (e.g., c.55G > C with a ratio of 1/21.72) may not be of pathogenic significance.

It's important to acknowledge the limitations of our FLGSA assay. For instance, like the minigene assay, our experiments required transfected cells, which may not always faithfully recapitulate in vivo conditions. However, findings from correlation of our FLGSA data with SpliceAI and cross-comparisons of our FLGSA data across different variants gave strong support to the validity of our FLGSA assay.

## Conclusions

By integrating the FLGSA assay with SpliceAI predictions, our study presents compelling evidence that 1.67% of potential *SPINK1* coding SNVs exert a discernible impact on splicing outcomes. Our findings underscore the critical necessity of conducting splicing analysis within the broader genomic context of the target gene, a perspective that can reveal splicing outcomes often missed by conventional minigene assays. Additionally, we emphasize the inherent uncertainties associated with intermediate SpliceAI scores (ranging from 0.20 to 0.80), highlighting the critical role of functional analysis in variant interpretation. Finally, our approach offers potential implications for transitioning from "retrospective" to "prospective" variant analysis in other disease genes, accelerating the full realization of precision medicine in the exome sequencing or genome sequencing era.

### Supplementary Information

**Additional file 1: Table S1.** SpliceAI predictions for all possible SPINK1 coding SNVs.

**Additional file 2: Figure S1.** Full-length gel images for Figure 6.

**Additional file 3: Figure S2.** Full-length gel image for Figure 10.

## Data Availability

All supporting data are available within the article.

## Abbreviations

ACMG: The American College of Medical Genetics and Genomics
AG: Acceptor gain
AL: Acceptor loss
DG: Donor gain
DL: Donor loss
FLGSA: Full-length gene splicing assay
HEK293T cells: Human embryonic kidney 293 T cells
MAVE: Multiplexed assays for variant effects
PWM: Position weight matrices
RT: Reverse transcription
RT-PCR: Reverse transcription-PCR
SNVs: Single-nucleotide variants
WT: Wild-type

## References

[1] Lim KH, Ferraris L, Filloux ME, Raphael BJ, Fairbrother WG (2011). Using positional distribution to identify splicing elements and predict pre-mRNA processing defects in human genes. Proc Natl Acad Sci U S A.

[2] Manning KS, Cooper TA (2017). The roles of RNA processing in translating genotype to phenotype. Nat Rev Mol Cell Biol.

[3] Cartegni L, Chew SL, Krainer AR (2002). Listening to silence and understanding nonsense: exonic mutations that affect splicing. Nat Rev Genet.

[4] Sarkar A, Panati K, Narala VR (2022). Code inside the codon: The role of synonymous mutations in regulating splicing machinery and its impact on disease. Mutat Res Rev Mutat Res.

[5] Aicher JK, Jewell P, Vaquero-Garcia J, Barash Y, Bhoj EJ (2020). Mapping RNA splicing variations in clinically accessible and nonaccessible tissues to facilitate Mendelian disease diagnosis using RNA-seq. Genet Med.

[6] Wai HA, Lord J, Lyon M, Gunning A, Kelly H, Cibin P (2020). Blood RNA analysis can increase clinical diagnostic rate and resolve variants of uncertain significance. Genet Med.

[7] Gaildrat P, Killian A, Martins A, Tournier I, Frebourg T, Tosi M (2010). Use of splicing reporter minigene assay to evaluate the effect on splicing of unclassified genetic variants. Methods Mol Biol.

[8] Wu H, Boulling A, Cooper DN, Li ZS, Liao Z, Chen JM (2017). In vitro and in silico evidence against a significant effect of the *SPINK1* c.194G>A variant on pre-mRNA splicing. Gut.

[9] Lin JH, Wu H, Zou WB, Masson E, Fichou Y, Le Gac G (2021). Splicing outcomes of 5' splice site GT>GC variants that generate wild-type transcripts differ significantly between full-length and minigene splicing assays. Front Genet.

[10] Fu XD, Ares M (2014). Context-dependent control of alternative splicing by RNA-binding proteins. Nat Rev Genet.

[11] Drexler HL, Choquet K, Churchman LS (2020). Splicing kinetics and coordination revealed by direct nascent RNA sequencing through nanopores. Mol Cell.

[12] Jaganathan K, Kyriazopoulou Panagiotopoulou S, McRae JF, Darbandi SF, Knowles D, Li YI (2019). Predicting splicing from primary sequence with deep learning. Cell.

[13] Lord J, Baralle D (2021). Splicing in the diagnosis of rare disease: advances and challenges. Front Genet.

[14] Dawes R, Joshi H, Cooper ST (2022). Empirical prediction of variant-activated cryptic splice donors using population-based RNA-Seq data. Nat Commun.

[15] Masson E, Zou WB, Pu N, Rebours V, Genin E, Wu H (2023). Classification of *PRSS1* variants responsible for chronic pancreatitis: An expert perspective from the Franco-Chinese GREPAN study group. Pancreatology.

[16] Walker LC, Hoya M, Wiggins GAR, Lindy A, Vincent LM, Parsons MT (2023). Using the ACMG/AMP framework to capture evidence related to predicted and observed impact on splicing: recommendations from the ClinGen SVI Splicing Subgroup. Am J Hum Genet.

[17] Zhang G, Hu Y, Yang Q, Pu N, Li G, Zhang J (2023). Frameshift coding sequence variants in the *LPL* gene: identification of two novel events and exploration of the genotype-phenotype relationship for variants reported to date. Lipids Health Dis.

[18] Richter F, Rutherford KD, Cooke AJ, Meshkati M, Eddy-Abrams V, Greene D (2024). A deep intronic *PKHD1* variant identified by SpliceAI in a deceased neonate with autosomal recessive polycystic kidney disease. Am J Kidney Dis.

[19] Inoue Y, Tsuchida N, Kim CA, de Oliveira SB, Castro MAA, Honjo RS (2024). Novel compound heterozygous *ABCA2* variants cause IDPOGSA, a variable phenotypic syndrome with intellectual disability. J Hum Genet.

[20] Richards S, Aziz N, Bale S, Bick D, Das S, Gastier-Foster J (2015). Standards and guidelines for the interpretation of sequence variants: a joint consensus recommendation of the American College of Medical Genetics and Genomics and the Association for Molecular Pathology. Genet Med.

[21] Shendure J, Findlay GM, Snyder MW (2019). Genomic medicine-progress, pitfalls, and promise. Cell.

[22] Starita LM, Ahituv N, Dunham MJ, Kitzman JO, Roth FP, Seelig G (2017). Variant interpretation: functional assays to the rescue. Am J Hum Genet.

[23] Gasperini M, Starita L, Shendure J (2016). The power of multiplexed functional analysis of genetic variants. Nat Protoc.

[24] Findlay GM, Daza RM, Martin B, Zhang MD, Leith AP, Gasperini M (2018). Accurate classification of *BRCA1* variants with saturation genome editing. Nature.

[25] Witt H, Luck W, Hennies HC, Classen M, Kage A, Lass U (2000). Mutations in the gene encoding the serine protease inhibitor, Kazal type 1 are associated with chronic pancreatitis. Nat Genet.

[26] Rosendahl J, Landt O, Bernadova J, Kovacs P, Teich N, Bodeker H (2013). *CFTR*, *SPINK1*, *CTRC* and *PRSS1* variants in chronic pancreatitis: is the role of mutated *CFTR* overestimated?. Gut.

[27] Masson E, Chen JM, Audrezet MP, Cooper DN, Férec C (2013). A conservative assessment of the major genetic causes of idiopathic chronic pancreatitis: data from a comprehensive analysis of *PRSS1*, *SPINK1*, *CTRC* and *CFTR* genes in 253 young French patients. PLoS ONE.

[28] Zou WB, Tang XY, Zhou DZ, Qian YY, Hu LH, Yu FF (2018). *SPINK1*, *PRSS1*, *CTRC*, and *CFTR* genotypes influence disease onset and clinical outcomes in chronic pancreatitis. Clin Transl Gastroenterol.

[29] Yamamoto T, Nakamura Y, Nishide J, Emi M, Ogawa M, Mori T (1985). Molecular cloning and nucleotide sequence of human pancreatic secretory trypsin inhibitor (*PSTI*) cDNA. Biochem Biophys Res Commun.

[30] Horii A, Kobayashi T, Tomita N, Yamamoto T, Fukushige S, Murotsu T (1987). Primary structure of human pancreatic secretory trypsin inhibitor (*PSTI*) gene. Biochem Biophys Res Commun.

[31] Hegyi E, Sahin-Tóth M (2017). Genetic risk in chronic pancreatitis: the trypsin-dependent pathway. Dig Dis Sci.

[32] Masson E, Zou WB, Genin E, Cooper DN, Le Gac G, Fichou Y (2022). Expanding ACMG variant classification guidelines into a general framework. Hum Genomics.

[33] Boulling A, Chen JM, Callebaut I, Férec C (2012). Is the *SPINK1* p.Asn34Ser missense mutation per se the true culprit within its associated haplotype?. WebmedCentral. GENETICS..

[34] Zou WB, Boulling A, Masson E, Cooper DN, Liao Z, Li ZS (2016). Clarifying the clinical relevance of *SPINK1* intronic variants in chronic pancreatitis. Gut.

[35] Zou WB, Masson E, Boulling A, Cooper DN, Li ZS, Liao Z (2016). Digging deeper into the intronic sequences of the *SPINK1* gene. Gut.

[36] Zou WB, Wu H, Boulling A, Cooper DN, Li ZS, Liao Z (2017). In silico prioritization and further functional characterization of *SPINK1* intronic variants. Hum Genomics.

[37] Tang XY, Lin JH, Zou WB, Masson E, Boulling A, Deng SJ (2019). Toward a clinical diagnostic pipeline for *SPINK1* intronic variants. Hum Genomics.

[38] Wu H, Boulling A, Cooper DN, Li ZS, Liao Z, Férec C (2017). Analysis of the impact of known *SPINK1* missense variants on pre-mRNA splicing and/or mRNA stability in a full-length gene assay. Genes (Basel).

[39] Lin JH, Tang XY, Boulling A, Zou WB, Masson E, Fichou Y (2019). First estimate of the scale of canonical 5' splice site GT>GC variants capable of generating wild-type transcripts. Hum Mutat.

[40] Kume K, Masamune A, Kikuta K, Shimosegawa T (2006). [-215G>A; IVS3+2T>C] mutation in the *SPINK1* gene causes exon 3 skipping and loss of the trypsin binding site. Gut.

[41] Chen JM, Lin JH, Masson E, Liao Z, Férec C, Cooper DN (2020). The experimentally obtained functional impact assessments of 5' splice site GT>GC variants differ markedly from those predicted. Curr Genomics.

[42] Ota Y, Masamune A, Inui K, Kume K, Shimosegawa T, Kikuyama M (2010). Phenotypic variability of the homozygous IVS3+2T>C mutation in the serine protease inhibitor Kazal type 1 (*SPINK1*) gene in patients with chronic pancreatitis. Tohoku J Exp Med.

[43] Venet T, Masson E, Talbotec C, Billiemaz K, Touraine R, Gay C (2017). Severe infantile isolated exocrine pancreatic insufficiency caused by the complete functional loss of the *SPINK1* gene. Hum Mutat.

[44] Wu H, Lin JH, Tang XY, Zou WB, Schutz S, Masson E, Fichou F, Le Gac G, Férec C, Liao Z, Chen JM. medRxiv 2023.11.14.23298498; doi: 10.1101/2023.11.14.23298498

[45] Morales J, Pujar S, Loveland JE, Astashyn A, Bennett R, Berry A (2022). A joint NCBI and EMBL-EBI transcript set for clinical genomics and research. Nature.

[46] Illumina precomputed SpliceAI scores. https://github.com/Illumina/SpliceAI (version 1.3). Accessed 18 February 2020.

[47] SpliceAI Virtual website. https://mobidetails.iurc.montp.inserm.fr/MD. Accessed 29 September 2023.

[48] SpliceAI Lookup. https://spliceailookup.broadinstitute.org/. Accessed 16 October 2023.

[49] ImageJ. https://imagej.net/. Accessed 18 October 2023.

[50] ChatGPT-4. https://chat.openai.com/. Last accessed 05 February 2024.

[51] SPINK1. https://www.ncbi.nlm.nih.gov/gene/6690. Accessed 16 October 2023.

[52] Leman R, Gaildrat P, Gac GL, Ka C, Fichou Y, Audrezet MP (2018). Novel diagnostic tool for prediction of variant spliceogenicity derived from a set of 395 combined in silico/in vitro studies: an international collaborative effort. Nucleic Acids Res.

[53] Leman R, Parfait B, Vidaud D, Girodon E, Pacot L, Le Gac G (2022). SPiP: Splicing Prediction Pipeline, a machine learning tool for massive detection of exonic and intronic variant effects on mRNA splicing. Hum Mutat.

[54] Lord J, Oquendo CJ, Wai HA, Douglas AGL, Bunyan DJ, Wang Y (2024). Predicting the impact of rare variants on RNA splicing in CAGI6. Hum Genet.

[55] Dawes R, Bournazos AM, Bryen SJ, Bommireddipalli S, Marchant RG, Joshi H (2023). SpliceVault predicts the precise nature of variant-associated mis-splicing. Nat Genet.

[56] Riepe TV, Khan M, Roosing S, Cremers FPM, Hoen PAC (2021). Benchmarking deep learning splice prediction tools using functional splice assays. Hum Mutat.

[57] Smith C, Kitzman JO (2023). Benchmarking splice variant prediction algorithms using massively parallel splicing assays. Genome Biol.

[58] de Sainte Agathe JM, Filser M, Isidor B, Besnard T, Gueguen P, Perrin A (2023). SpliceAI-visual: a free online tool to improve SpliceAI splicing variant interpretation. Hum Genomics.

