# Supplementary material for: Combining full-length gene assay and SpliceAI to interpret the splicing impact of all possible SPINK1 coding variants
Source: Hum Genomics. 2024 Feb 27;18:21. doi: 10.1186/s40246-024-00586-9 (PMC10898081; doi:10.1186/s40246-024-00586-9)
Supplement: Supplementary file 2 — Additional file 2: Figure S1. Full-length gel images for Figure 6. [file 40246_2024_586_MOESM2_ESM.ppt]

## Slide 1
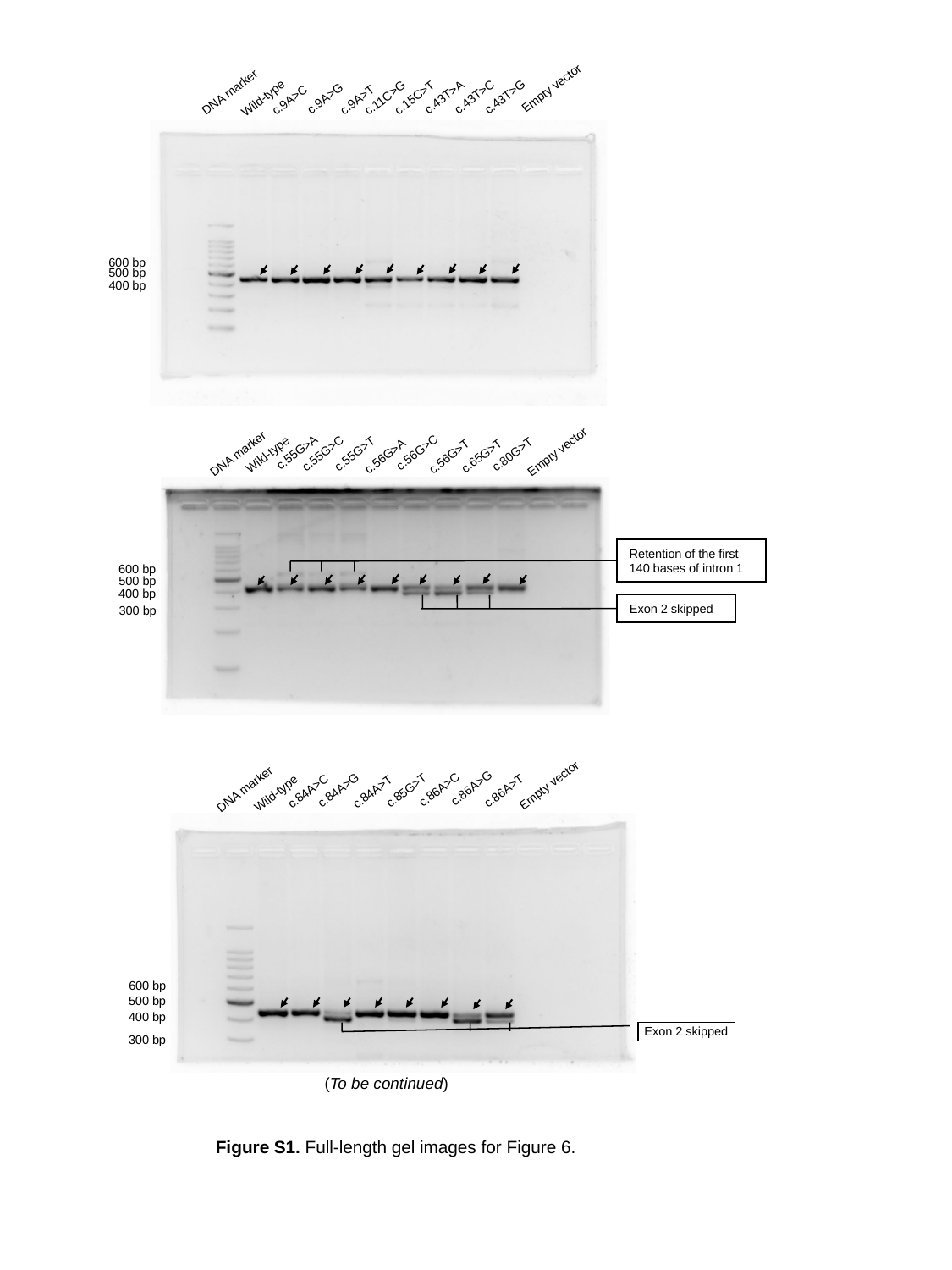

Empty vector
 DNA marker
 Wild-type
 c.9A>G
 c.43T>A
 c.43T>C
 c.43T>G
 c.9A>T
 c.15C>T
 c.9A>C
 c.11C>G
600 bp
500 bp
400 bp
 Wild-type
 c.55G>A
 Empty vector
 DNA marker
 c.56G>C
 c.55G>C
 c.55G>T
 c.80G>T
 c.65G>T
 c.56G>A
 c.56G>T
Retention of the first 140 bases of intron 1
600 bp
500 bp
400 bp
Exon 2 skipped
300 bp
 Empty vector
 DNA marker
 c.86A>G
 c.86A>C
 c.84A>G
 c.85G>T
 c.86A>T
 Wild-type
 c.84A>C
 c.84A>T
600 bp
500 bp
400 bp
Exon 2 skipped
300 bp
(To be continued)
Figure S1. Full-length gel images for Figure 6.

## Slide 2
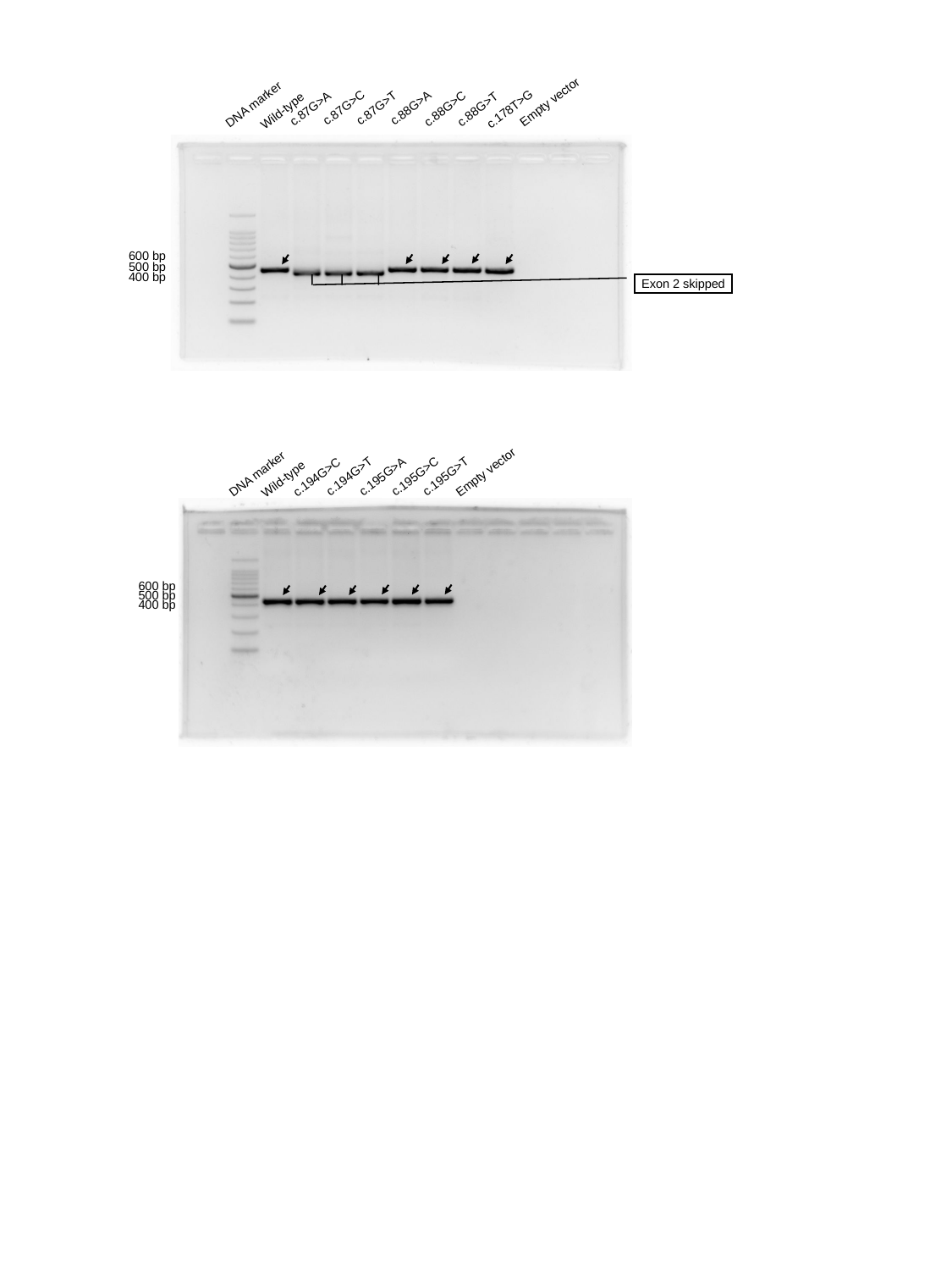

Empty vector
 DNA marker
 c.87G>C
 c.87G>T
 c.88G>A
 Wild-type
 c.87G>A
 c.88G>C
 c.88G>T
c.178T>G
600 bp
500 bp
400 bp
Exon 2 skipped
 DNA marker
 Empty vector
c.195G>C
c.194G>C
 Wild-type
c.194G>T
c.195G>A
c.195G>T
600 bp
500 bp
400 bp
