# Supplementary material for: Combining full-length gene assay and SpliceAI to interpret the splicing impact of all possible SPINK1 coding variants
Source: Hum Genomics. 2024 Feb 27;18:21. doi: 10.1186/s40246-024-00586-9 (PMC10898081; doi:10.1186/s40246-024-00586-9)
Supplement: Supplementary file 3 — Additional file 3: Figure S2. Full-length gel image for Figure 10. [file 40246_2024_586_MOESM3_ESM.ppt]

## Slide 1
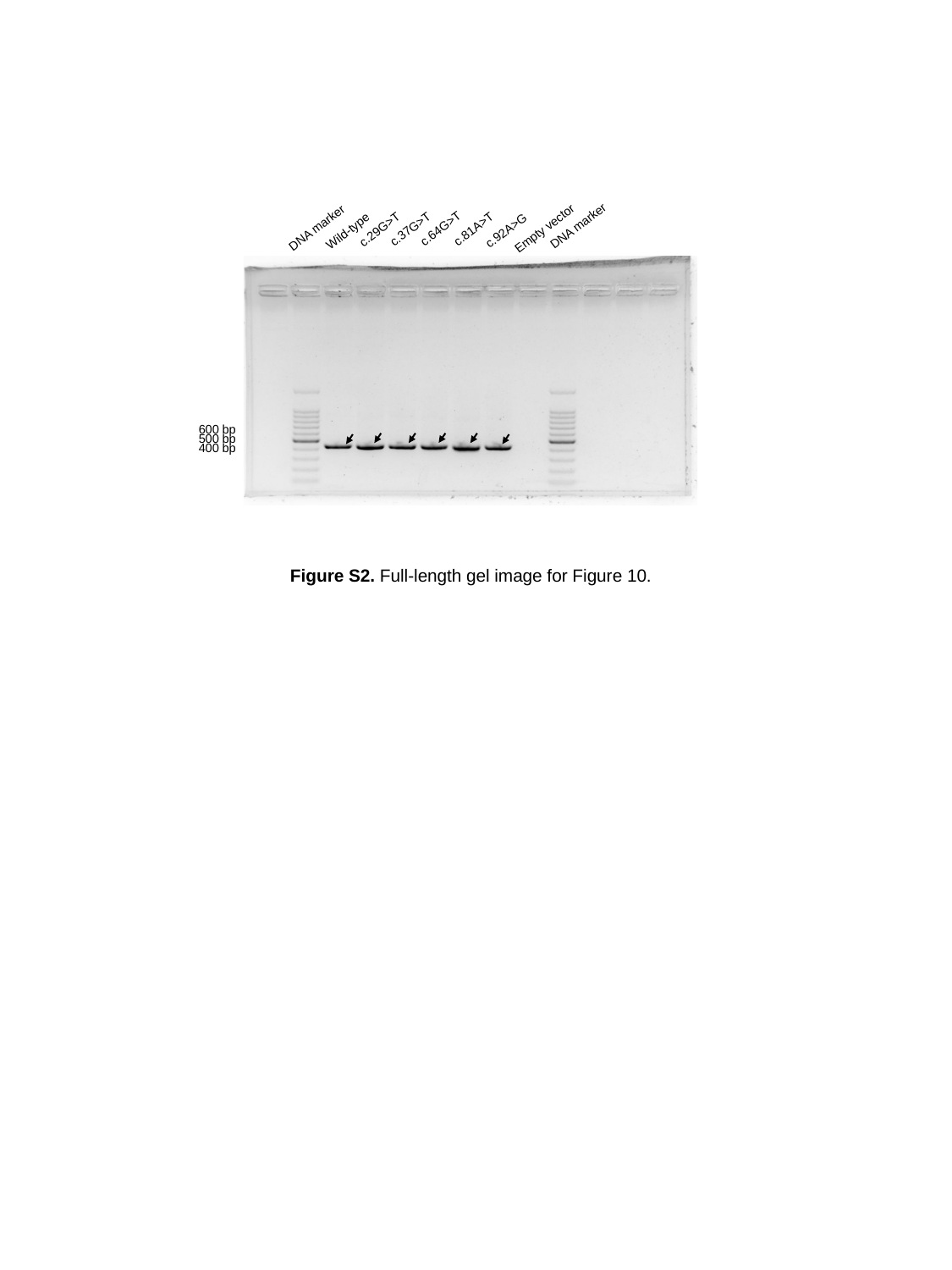

DNA marker
 DNA marker
c.81A>T
c.29G>T
 Wild-type
 Empty vector
c.64G>T
c.37G>T
c.92A>G
600 bp
500 bp
400 bp
Figure S2. Full-length gel image for Figure 10.
